# Anti-inflammatory and immunomodulatory effects of polysaccharide extracted from Wuguchong (maggot) on 2,4-dinitrochlorobenzene-induced atopic dermatitis in mice

**DOI:** 10.3389/fphar.2023.1119103

**Published:** 2023-03-22

**Authors:** Fangli Peng, Junwei Zong, Tianqi Zhao, Peng Shi, Ming Lu, Xueling Qu, Xin Han, Lin Zhao, Zhuqiang Jia, Shouyu Wang

**Affiliations:** ^1^ Department of Orthopaedic Surgery, The First Affiliated Hospital of Dalian Medical University, Dalian, China; ^2^ College of Integrative Medicine, Dalian Medical University, Dalian, China; ^3^ Department of Trauma and Tissue Repair Surgery, Dalian Municipal Central Hospital, Dalian, China; ^4^ Changjianglu Pelvic Floor Repair Center, Dalian Women and Children’s Medical Group, Dalian, China; ^5^ Department of Orthopaedic Surgery, The Second Affiliated Hospital of Dalian Medical University, Dalian, China; ^6^ Naqu People’s Hospital, Tibet, China; ^7^ Department of Quality Management, Dalian Municipal Central Hospital, Dalian, China; ^8^ The First Affiliated Hospital of Dalian Medical University, Dalian, China

**Keywords:** atopic dermatitis, polysaccharide, Wuguchong, CD4^+^ T cells, anti-inflammation, immunomodulation

## Abstract

Atopic dermatitis (AD) is an inflammatory, heterogeneous, chronic skin disorder characterized by recurrent eczematous lesions and intense pruritus, and the pathophysiology mechanism of AD is known for immune dysregulation and inflammatory responses. Wuguchong (maggot) has been widely used in the wound field and found with pharmacological properties of the anti-inflammatory and immunomodulatory function. Recently, some polysaccharides were proven to have beneficial effects on AD skin lesions in mice and humans. However, the effect of the polysaccharide extracted from Wuguchong (PEW) on AD remains to be investigated. In the present study, we examined the anti-inflammatory and immunomodulatory effects of PEW on AD and explored the potential mechanisms. Balb/c mice were orally administrated with PEW to evaluate the therapeutic effect of PEW on 2,4-dinitrochlorobenzene (DNCB)-induced AD. Oral PEW administration significantly ameliorated the lesions and symptoms in AD mice, such as the ear thickness and ear swelling degree, epidermal and dermal thickness, and the infiltration of mast cells. In addition, PEW treatment decreased the levels of serum IgE and histamine, the frequencies of Th1 and Th17 cells, as well as the mRNA expression levels of Th1 and Th17 cytokines and nuclear transcript factors (IFN-γ, T-bet, IL-17A, and ROR-rt). Furthermore, the activation of the NF-κB pathway and the phosphorylation of MAPKs (p38, ERK, and JNK) were significantly suppressed by PEW treatment. Taken together, our study suggests that PEW exerts anti-inflammatory and immunomodulatory effects through inhibition of Th1 and Th17 responses and downregulation of NF-κB and MAPK pathways, PEW would be developed as a promising immune therapy for AD.

## 1 Introduction

Atopic dermatitis (AD), also known as eczema and atopic eczema, is a chronic, heterogeneous, inflammatory skin disorder characterized by intense itching and recurrent eczematous lesions and has wide-ranging clinical phenotypes and courses (Langan et al., 2020; Bieber et al., 2022). The prevalence and incidence of atopic dermatitis have increased over the past several decades (Asher et al., 2006; Nutten, 2015), affecting 15%–20% of children and up to 10% of adults (Drucker et al., 2018). Interactions among genetic and environmental factors, skin barrier dysfunction, immune dysregulation, microbial imbalance, and environmental triggers of skin inflammation contribute to the pathogenesis of atopic dermatitis (Luger et al., 2021; Patrick et al., 2021; Ständer, 2021). The treatments of atopic dermatitis include barrier repair and maintenance therapy (moisturizers), topical anti-inflammatory therapies (topical corticosteroids, topical calcineurin inhibitors, novel phosphodiesterase 4 inhibitors), conventional systemic treatments (glucocorticoids, cyclosporine, or methotrexate), JAK inhibitors (topical and systemic), phototherapy, targeted monoclonal antibodies (Th2-targeted therapies have been well-developed, monoclonal interleukin-4, −13, −22, and −31 receptor antibodies are promising agents) (Langan et al., 2020; Luger et al., 2021; Ständer, 2021). However, current therapies for AD face grim challenges, such as serious side effects, adverse events and drug tolerance of antibiotic and corticosteroid therapy, long-term and functionally impairing conjunctivitis with IL-4 receptor antibody, the risk of thromboembolism and cancer of JAK inhibitors, the high cost of phototherapy, etc. (Cameron et al., 2014; Ständer, 2021); moreover, a considerable number of patients are non-responders to the available regimens (Nogueira and Torres, 2021). AD inflicts a heavy socio-economic burden (Langan et al., 2020; Laughter et al., 2021), partly due to the lack of long-term safe effective therapeutics. This leads to an urgent need to develop alternative and complementary medicine as a new therapeutic option for AD. Traditional Chinese medicine (TCM) has long been widely used as routine management and treatment for AD, including many formulas and biologically active ingredients (Hon et al., 2011; Yan et al., 2020). Recently, increasing evidence has demonstrated that some polysaccharides are effective in alleviating AD-like skin lesions in mice, such as polysaccharide extract from phragmites rhizome (Nam et al., 2013), black currant (Ribes nigrum L.) (Ashigai et al., 2018), Chinese white wax scale (Lin et al., 2017), Aphanothece Sacrum (Motoyama et al., 2018), and Fucoidan (Tian et al., 2019), etc. In addition, an 8-week clinical study demonstrated oral systemic treatment with the polysaccharide extract from Dendrobium huoshanense had significant beneficial effects on AD symptoms in children (Wu et al., 2011).

CD4^+^ T cells are the dominant cellular infiltration in AD and are differentiated into Th1, Th2, Th17, Th22, and regulatory T cell subsets (Bieber et al., 2022), increased frequencies of Th1, Th2, Th17, and Th22 cells together with an excessive accumulation of their inflammatory cytokines play vital roles in AD pathogenesis (Grewe et al., 1998; Weidinger and Novak, 2016). AD is historically considered a bipolar T-cell-mediated disease, in which the acute phase is predominated by the Th2 signal, with a Th2-to-Th1 shift to promote disease chronicity (Thepen et al., 1996; Furue et al., 2017). Nevertheless, a recent study proved that acute AD is induced by both Th2 and Th22 activation, and these pathways show an intensification in chronic AD, rather than a simple switch to a primarily Th1 response in the chronic phase (Gittler et al., 2012; Mansouri and Guttman-Yassky, 2015). In acute AD lesions, Th2 cytokines (IL-4, IL-31, and IL-13) and Th22 cytokine (IL-22) are markedly accumulated (Gittler et al., 2012; Mansouri and Guttman-Yassky, 2015). As the disease progresses to the chronic phase, further significant accumulations are seen in Th2-related (IL-13, IL-5, IL-10, IL-31, CCL5, CCL13, and CCL18) and Th22-related molecules (IL-32 and S100A7-9), as well as Th1-related products (INF-γ, IL-1β, CXCL 9–11, and MX1) (Gittler et al., 2012; Mansouri and Guttman-Yassky, 2015). Whereas Th17 activation shows similar magnitude in chronic and acute AD, without further intensification (Gittler et al., 2012; Mansouri and Guttman-Yassky, 2015). Th17 pathways are activated in certain subtypes such as pediatric, intrinsic, and Asian-origin AD (Yu et al., 2019). Th17 cells were proven to be directly related to AD severity (Koga et al., 2008). Although Th17 cell frequency is not closely associated with the Th1/Th2 balance, there is a significant correlation between the percentages of IL-17^+^ and INF-γ^+^ cells (Koga et al., 2008). Even though IL-17 cells were known to produce a small amount of IL-22 cytokine, a distinct T cell subset—Th22 cell, has recently been identified to produce IL-22 and is related to epidermal immunity and remodeling (Duhen et al., 2009; Eyerich et al., 2009; Mirshafiey et al., 2015). Upregulated IL-22 causes epidermal acanthosis in chronic AD skin (Nograles et al., 2009). IL-22 plays vital roles in AD initiation, development, and severity, partly by inducing epithelial Th2 cytokines production and the GRP pathway activation (Nograles et al., 2009; Lou et al., 2017).

The nuclear factor-κB (NF-κB) pathway plays important roles in immunity and inflammation, deregulated NF-κB activation contributes to the pathogenic processes of various inflammatory diseases including AD (Oh and Ghosh, 2013; Liu et al., 2017; Ko et al., 2022). The canonical NF-κB pathway regulates the differentiation of CD4^+^ T cells through both regulation of cytokine production in innate immune cells and T-cell intrinsic mechanisms (Oh and Ghosh, 2013; Liu et al., 2017). In addition, the mitogen-activated protein kinase (MAPK) cascade, which includes the p38, c-jun N-terminal kinase (JNK), and extracellular signal-regulated kinase (ERK) MAPK, is also an important signaling pathway in immune responses and cross talks with NF-κB to regulate inflammation signaling (Ko et al., 2022). Phosphorylation of MAPKs leads to inflammatory mediators’ production and promotes the allergic inflammatory response (Barnes, 2011; Arthur and Ley, 2013). And the MAPK pathways are involved in the pathogenesis of inflammatory skin diseases, including AD (Johansen et al., 2005; Lu et al., 2018; Lee et al., 2022; Zeze et al., 2022).

Total serum IgE level has been reported to be correlated with AD and the severity of the disease, which is a possible marker of AD activity (Dhar et al., 2005). The IgE-mast cell axis is known to play an important role in lots of allergic and inflammatory responses (Stone et al., 2010; Galli and Tsai, 2012). A majority of AD skin lesions see an increase in mast cells (Kawakami et al., 2009). In mast cells, degranulation, generation, and secretion of allergic mediators like histamine, some pro-inflammatory cytokines, and many proteases are realized *via* binding their high-affinity surface receptors to IgE (Theoharides et al., 2012). Among the allergic mediators released by mast cells, histamine enhances the production of some pro-inflammatory cytokines and chemokines, thus contributing to the progression of allergic-inflammatory responses (Jutel et al., 2009).

Wuguchong (Maggot), the larvae of *Lucilia sericata*, which belongs to the family Calliphoridae within the order Diptera, is a traditional Chinese medicine widely used in the wound field (Yan et al., 2018). Pharmacological properties of the Wuguchong include antibacterial activity, anti-inflammatory activity, immunomodulatory function, proangiogenic activity, neurogenesis, etc (Yan et al., 2018). However, poor acceptance by both patients and healthcare professionals is a major obstacle to the utilization of maggot therapy; the so-called ‘yuk’ factor or social and cultural beliefs may initially hinder its use (Richardson, 2004). So, more and more researchers are trying to extract active ingredients from Wuguchong (maggot) to improve its clinical utilization and acceptance. Recently, the polysaccharide extracted from Wuguchong (PEW) was found to be useful in reducing high-fat diet-induced obesity in mice through regulation of the intestinal microbiota composition (Wang et al., 2020). Besides, homogeneous polysaccharides of Wuguchong showed a protective effect on the intestinal mucosa and can relieve 5-fluorouracil-induced intestinal inflammation in mice (Shi et al., 2022). As previously discussed, some polysaccharides were proven to be effective in ameliorating AD symptoms in mice or humans. However, currently, it is unknown whether the polysaccharide extracted from Wuguchong (PEW) is effective in treating AD. In this study, the effect of PEW on DNCB-induced AD in Balb/c mice and the underlying mechanisms involved in immunoregulation and anti-inflammation were evaluated through the analysis of CD4^+^ Th cell subset: Th1, Th2, Th17, Th22 cells, and their related cytokines and nuclear transcription factors levels. Our findings indicate that PEW relieves AD-like symptoms in mice *via* inhibiting Th1 and Th17 responses and downregulating NF-κB and MAPK pathways.

## 2 Materials and methods

### 2.1 PEW preparation

The PEW was isolated from the dried bodies of Wuguchong according to water extraction and alcohol precipitation methods and characterized by GPC (gel permeation chromatogram) and HPLC (high-performance liquid chromatography). The molecular weight of PEW is 32.9 kDa, and its monosaccharide composition includes rhamnose, glucose, arabinose, mannose, xylose, galactose, galacturonic acid, and glucuronic acid as previously reported (Wang et al., 2020).

### 2.2 Animals and treatment

The present study was approved by the Ethical Committee on Animal Research of Dalian Medical University (approved number: AEE19075). Balb/c female mice (18 ± 2 g, 6–8 weeks) were bred in an animal facility under standard laboratory conditions in the SPF-level Experimental Animal Center of Dalian Medical University (Dalian, China).

After 1 week of acclimation, all mice were shaved about 2.5 cm × 2.5 cm on their dorsal skin on day 0 and then randomly divided into 5 groups (*n* = 9–10/group) as follows:

Group 1: physiological saline.

Group 2: DNCB + physiological saline.

Group 3: DNCB + PEW (1000 mg/kg.d) + physiological saline.

Group 4: DNCB + PEW (2000 mg/kg.d) + physiological saline.

Group 5: DNCB + dexamethasone (DEX, 1 mg/kg.d) + physiological saline.

The mice were orally administered with physiological saline, PEW (1000 mg/kg.d), PEW (2000 mg/kg.d), and dexamethasone (DEX) (1 mg/kg.d) from day 4 to day 14. PEW and dexamethasone were separately dissolved in 0.9% physiological saline to get 100 mg/mL PEW solution, 200 mg/mL PEW solution, and 0.1 mg/mL dexamethasone solution, respectively. Mice were administered by oral gavage daily at a dose volume of 0.01 mL/g using a plastic syringe (1 mL) equipped with a straight, stainless-steel, bulb-tipped gavage needle (20-gauge, 38 mm).

**FIGURE 1:**
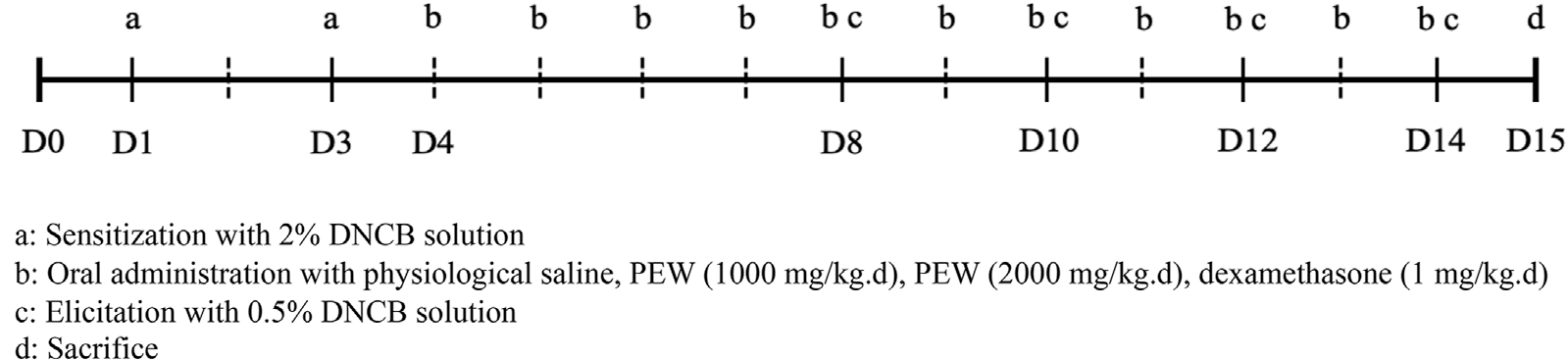
Schedule of the experiment. All mice were shaved about 2.5 cm × 2.5 cm on their dorsal skin on day 0. On day 1, the mice were first sensitized by painting with 120 μL of 2% DNCB solution on shaved dorsal skin and 25 μL on the back of the right ear. The same treatments were performed for the second sensitization on day 3. Four days later, the elicitation phase was induced by painting with 0.5% DNCB solution on the dorsal skin (120 μL) and the back of the right ear (25 μL) once every 2 days till day 14. All mice were sacrificed on day 15. Oral administration treatments were conducted from day 4 to day 14.

DNCB (Sigma-Aldrich, United States) was applied to the shaved dorsal skin and the back of the right ear of Balb/c mice to induce AD-like symptoms, based on the previously described procedure with minor modification (Lee et al., 2010; Lin et al., 2017). On day 1, the mice were first sensitized by painting with 120 μL of 2% DNCB solution (dissolved in a 3:1 mixture of acetone: olive oil) on shaved dorsal skin and 25 μL on the back of the right ear. The same treatments were performed on day 3 for the second sensitization. Four days later, 0.5% DNCB solution was painted on the dorsal skin (120 μL) and the back of the right ear (25 μL) to induce the elicitation phase, once every 2 days till day 14. All mice were sacrificed on day 15 (Figure 1).

### 2.3 Ear thickness and ear swelling degree

The ear thickness was measured on day 0 and 24 h after each DNCB application with a thickness gauge (Mitutoyo Corporation, Tokyo, Japan). Both ears of each mouse were punched with a 7 mm diameter skin puncher after the mice were sacrificed, the ear swelling degree was measured as the right ear weight minus the left ear weight.

### 2.4 Histological analysis

The dorsal skin tissues of mice were cut and fixed in 4% paraformaldehyde after being sacrificed. The fixed tissues were embedded in paraffin and then sliced into 4 µm sections. Hematoxylin and eosin (H&E) and toluidine blue (TB) were stained on the tissue sections for histological analysis. Epidermal thickness and dermal thickness were analyzed in H&E-stained sections at a magnification of ×100. Thickness was measured in five randomly selected fields from each sample. Toluidine blue (TB) stain for the measurement of mast cell infiltration and mast cell was counted in 5 random high-power fields at a magnification of ×400.

### 2.5 Enzyme-linked immunosorbent assay (ELISA)

Blood samples were collected immediately after the mice were sacrificed. The serum samples were obtained from blood using a centrifuge (3000 × g, 4°C, 15 min) and stored at −80°C until use. The level of serum IgE and serum histamine were measured by mouse IgE ELISA kit (Lengton Biological Technology, Shanghai, China) and mouse histamine kit (Elabscience Biotechnology, Wuhan, China) according to the manufacturer’s instructions, respectively.

### 2.6 Flow cytometry assay

Splenocytes were collected as single-cell suspension after the mice were sacrificed. The prepared splenocytes (5 × 10^6^) were cultured with the cell stimulation cocktail (plus protein transport inhibitors) (eBioscience, United States) for 6 h in a 5% CO_2_ incubator at 37°C. Then, the cells were collected and strained with FITC-labeled rat anti-mouse CD4 mAb (Clone RM4-5, BioLegend, United States) for 20 min at 4°C in the dark. The cells were fixed and permeabilized after being thoroughly resuspended in BD Cytofix/Cytoperm solution (BD Pharmingen, United States) for 20 min at 4°C and then washed with BD Perm/Wash™ buffer (BD Pharmingen, United States) two times. Next, the permeabilized cells were stained with PE-labeled rat anti-mouse IFN-γ mAb (Clone XMG1.2, BioLegend, United States), PE-Cy7-labeled rat anti-mouse IL-4 mAb (Clone 11B11, BioLegend, United States), PerCp-Cy5.5-labeled rat anti-mouse IL-17A mAb (Clone eBio17B7, eBioscience, United States) and APC-labeled rat anti-mouse IL-22 mAb (Clone Poly5164, BioLegend, United States) for 20 min at 4°C in the dark. Th1 cells were defined as CD4^+^ IFN-γ^+^, Th2 cells were defined as CD4^+^ IL-4^+^, Th17 cells were defined as CD4^+^ IL-17A^+^, and Th22 cells were defined as CD4^+^ IL-22^+^. Data was harvested with a BD FACSVerse Flow Cytometer (BD Pharmingen, United States) and analyzed using FlowJo software (BD, United States).

### 2.7 Real-time quantitative polymerase chain reaction (RT-qPCR)

The total RNA was isolated from mice’s dorsal skin tissue using TransZol (Transgen Biotech, Beijing, China) according to the manufacturer’s protocol. 10 μg of total RNA was used to synthesize the first-strand complementary DNA (cDNA) with the All-in-One First-Strand cDNA Synthesis kit (Transgen Biotech, Beijing, China). The relative mRNA expression was quantified using RT-qPCR with Top Green qPCR SuperMix (Transgen Biotech, Beijing, China) performed on an ABI Prism 7,500 device (Applied Biosystems, United States). Levels of target genes were normalized with respect to the expression of GAPDH, and the primer sequences used in this study are listed as follows (Table 1).

**TABLE 1:** The primer sequences.

Gene	Sequence (Forward)	Sequence (Reverse)	Size (bp)
T-bet	CTT​TGA​GTC​CAT​GTA​CGC​ATC​TGT	GGG​AAC​AGG​ATA​CTG​GTT​GGA​T	118
GATA-3	GAA​CTG​CGG​GGC​AAC​CTC​TA	TCC​CCA​TTA​GCG​TTC​CTC​CT	213
ROR-rt	TGT​AAT​GTG​GCC​TAC​TCC​TGC​A	GTA​TGT​AAG​TGT​GTC​TGC​TCC​GC	261
Ahr	CTT​CAT​CTT​CAG​GAC​CAA​ACA​CA	GAG​TGG​CGA​TGA​TGT​AAT​CTG​GT	296
IFN-γ	GCT​ACA​CAC​TGC​ATC​TTG​GCT	GGC​TTT​CAA​TGA​CTG​TGC​CG	82
IL-4	TAC​CAG​GAG​CCA​TAT​CCA​CGG​ATG	TGT​GGT​GTT​CTT​CGT​TGC​TGT​GAG	139
IL-17A	TCC​ACC​GCA​ATG​AAG​ACC​CT	CAT​GTG​GTG​GTC​CAG​CTT​TCC	104
Il-22	GCA​GAT​AAC​AAC​ACA​GAT​GTC​C	GTC​TTC​CAG​GGT​GAA​GTT​GAG	111
GAPDH	CCT​CGT​CCC​GTA​GAC​AAA​ATG	TGA​GGT​CAA​TGA​AGG​GGT​CGT	133

### 2.8 Western blotting

Total proteins were extracted from mice’s dorsal skin tissues using the Whole Cell Lysis Assay Kit (KeyGEN, Nanjing, China), followed by the protein concentrations determined with the BCA assay kit (Solarbio, Beijing, China). The denatured protein was separated by 10% SDS-PAGE and then transferred onto PVDF membranes (Millipore, United States). After being blocked with 5% BSA for 2 h at room temperature, the membranes were incubated overnight at 4°C with the following primary antibodies: NF-κB (p65), p-IκBα (Ser32), p-p38 (Thr180/Tyr182), p38, p-JNK (Thr183/Tyr185), JNK, p-ERK (Thr202/Tyr204), ERK (Cell Signaling Technology, United States) and GAPDH (Bioworld, Nanjing, China). Then, the HRP Goat Anti-Rabit IgG (H + L) secondary antibody (ABclonal, Wuhan, China) was added and incubated for 1 h at room temperature. The protein bands were visualized with the ECL FemtoLight kit (EpiZyme, Shanghai, China) and analyzed with the ImageJ software (NIH, Bethesda, MD, United States).

### 2.9 Statistical analysis

All statistical analyses were performed using Prism 8.0 (GraphPad Software, San Diego, CA, United States), and differences across groups were analyzed using unpaired Student’s *T*-test and one-way analysis of variance (ANOVA). The data were expressed as the mean ± standard deviation (SD), and *p* < 0.05 was considered statistically significant.

## 3 Results

### 3.1 PEW ameliorates DNCB-Induced AD-like phenotypic and histologic changes in mice

**FIGURE 2:**
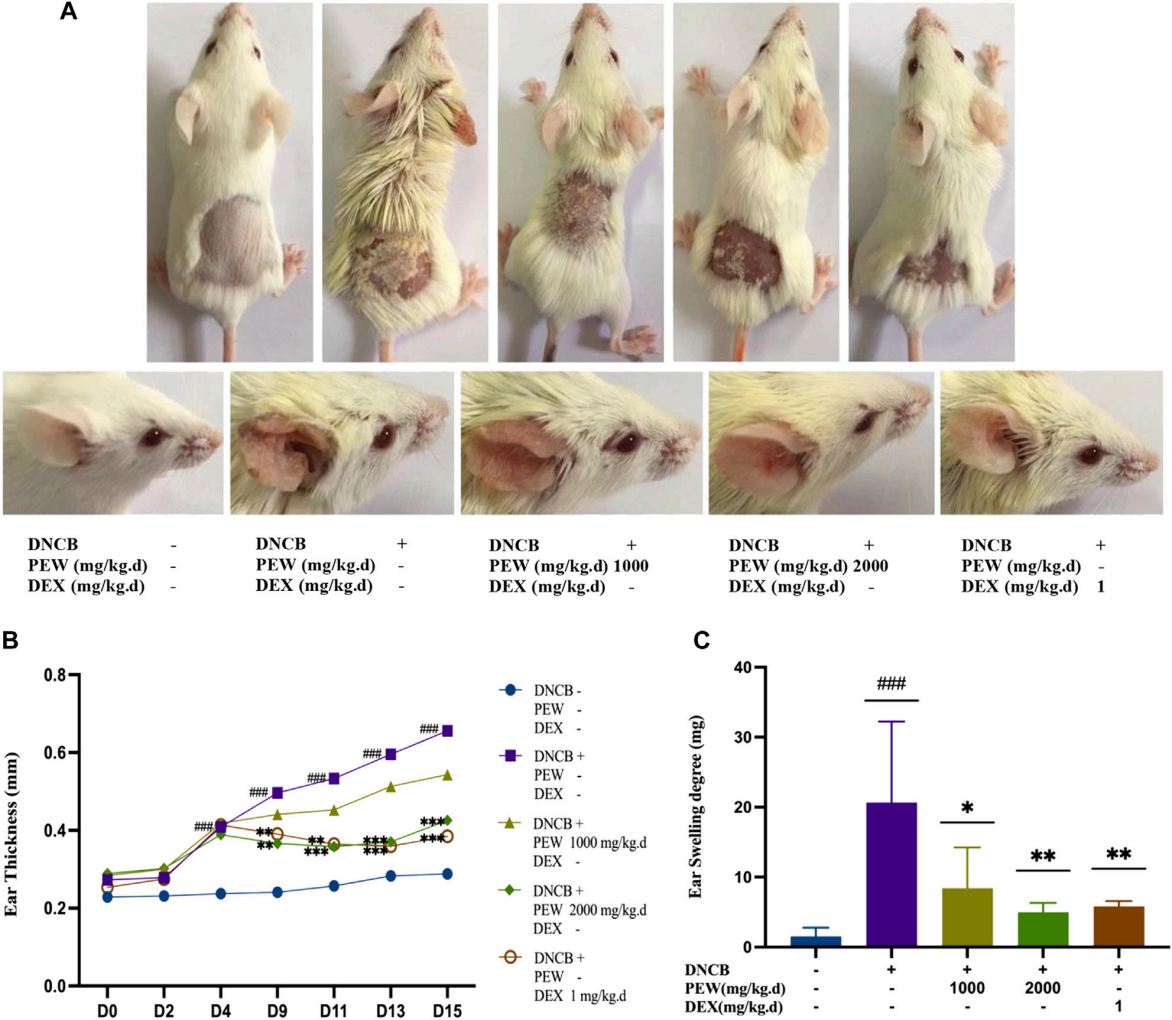
PEW ameliorates DNCB-Induced AD-like symptoms in Balb/c mice. **(A)**. Representative images of dorsal skins and ears in each group (day 15). **(B)** Changes in ear thickness were measured and recorded (*n* = 9–10/group). **(C)** The ear swelling degree was recorded as the right ear weight minus the left ear weight (a 7 mm punch) at the endpoint (*n* = 7–10/group). The data were presented as means ± SD. ### *p* < 0.001, VS. the normal group; * *p* < 0.05, ** *p* < 0.01, *** *p* < 0.001, VS. the model control group.

To explore the therapeutic properties of PEW on AD, a Balb/c AD model was established by applying DNCB on the dorsal skin and the back of the right ear (Figures 1, 2A). As shown in Figures 2B, C, oral administration of PEW (1000 mg/kg.d and 2000 mg/kg.d) significantly reduced the increase in ear thickness and ear swelling degree induced by DNCB in a dose-dependent manner.

**FIGURE 3:**
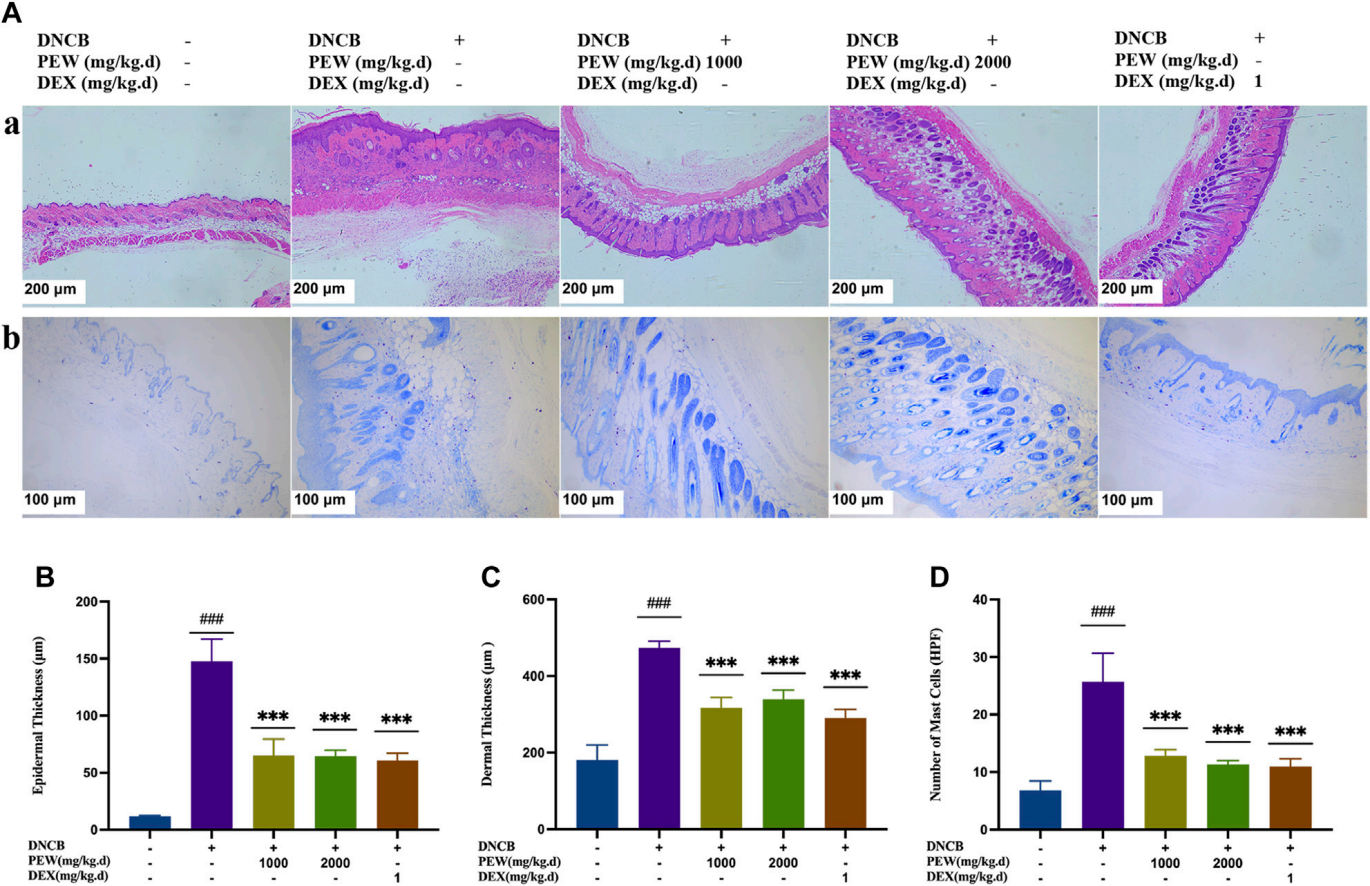
Effects of PEW on histopathological features of dorsal skin lesions in DNCB-induced Balb/c mice. **(A)** Representative images of histological examination (dorsal skin); (a) H&E staining (50×, scale bar = 200 μm); (b) toluidine blue staining (100×, scale bar = 100 μm). **(B)** Epidermal thickness (*n* = 5–6/group). **(C)** Dermal thickness (*n* = 5–6/group). **(D)** The number of mast cells (*n* = 5–6/group). The data were presented as means ± SD. ### *p* < 0.001, VS. the normal group; *** *p* < 0.001, VS. the model control group.

In Figure 3, H&E staining demonstrated that the PEW-treated groups (1000 mg/kg.d and 2000 mg/kg.d) showed significant decreases in the epidermal and dermal thickness (*p* < 0.001) (Figures 3A–C). In addition, PEW application significantly reduced the infiltration of mast cells labeled by toluidine blue in DNCB-induced AD mouse skin lesions in a dose-dependent manner (*p* < 0.001) [Figures 3A(b), D].

### 3.2 Effects of PEW on serum levels of IgE and histamine

**FIGURE 4:**
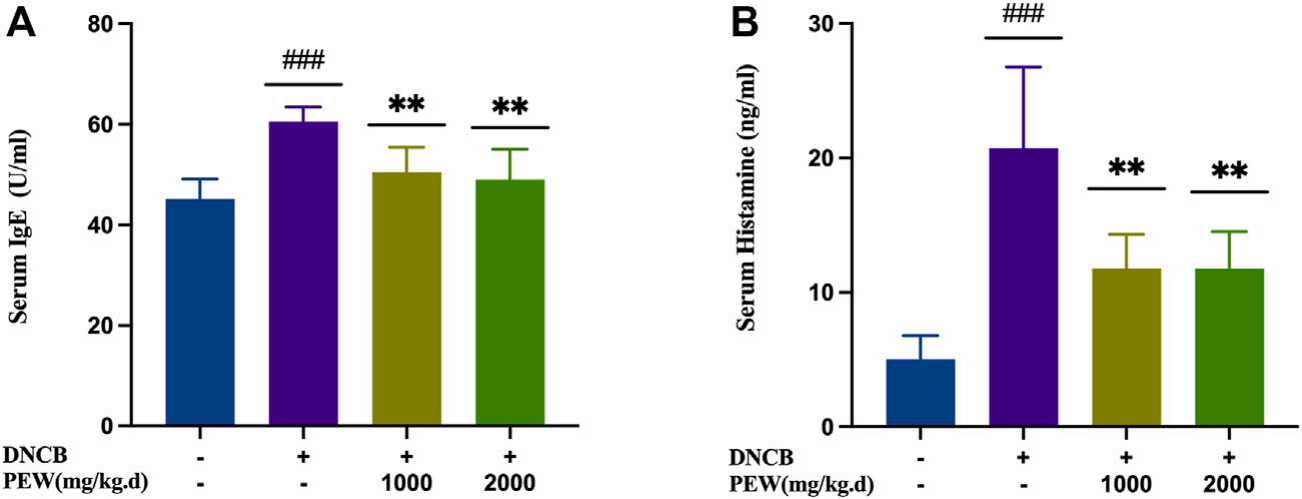
Effects of PEW on serum levels of IgE and histamine in DNCB-induced AD mice. **(A)** Levels of IgE in serum (*n* = 6/group). **(B)** Levels of histamine in serum (*n* = 6/group). The data were presented as means ± SD. ### *p* < 0.001, VS. the normal group; ** *p* < 0.01, VS. the model control group.

To evaluate the effects of PEW on the production of IgE and histamine in DNCB-induced AD mice, IgE and histamine levels were measured in the serum with ELISA kits. The AD model group showed significant increases in IgE and histamine levels compared with the normal control group (*p* < 0.001), whereas the PEW-treated groups (1000 mg/kg.d and 2000 mg/kg.d) displayed significantly lower levels than the AD model group (*p* < 0.01) (Figure 4).

### 3.3 PEW decreases the percentage of Th1 and Th17 cells in DNCB-induced AD mice

**FIGURE 5:**
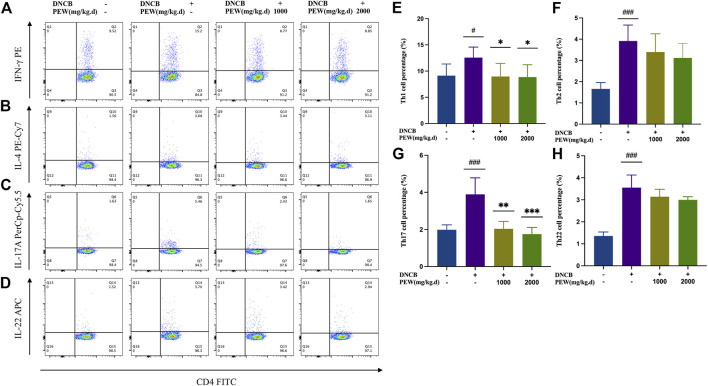
PEW decreases the percentage of Th1 and Th17 cells in DNCB-induced AD mice. Splenocytes were collected as single-cell suspension after the mice were sacrificed. The influence of PEW on the differentiation of CD4^+^ Th cells in DNCB-induced AD mice was evaluated by detecting the intracellular expression of IFN-γ, IL-4, IL-17A, and IL-22 using the Flow cytometry assay (*n* = 5–7/group). **(A–D)**. Representative images of different Th cell frequencies: **(A)** Th1, **(B)** Th2, **(C)** Th17, **(D)** Th22. **(E–G)**. Statistical analysis showed the differences in the percentages of **(E)** Th1, **(F)** Th2, **(G)** Th17, and **(H)** Th22 cells. The data were presented as means ± SD. # *p* < 0.05, ### *p* < 0.001, VS. the normal group; * *p* < 0.05, ** *p* < 0.01, *** *p* < 0.001, VS. the model control group.

Considering the relevance of Th1, Th2, Th17, and Th22 axes to AD immunopathogenesis, we evaluated the influence of PEW on the differentiation of CD4^+^ Th cells in DNCB-induced AD mice by detecting the intracellular expression of IFN-γ, IL-4, IL-17A, and IL-22 using Flow cytometry assay. In the present study, the percentage of Th1, Th2, Th17, and Th22 cells of the AD model group were all significantly increased compared to the normal control group. Two PEW-treated groups showed significantly lower percentages of Th1 and Th17 cell subsets than the AD model group (Figure 5). However, the percentage of Th2 and Th22 cell subsets exhibited no significant differences between the PEW-treated groups and the AD model group (Figure 5).

### 3.4 PEW reduces mRNA expression of T-bet, IFN-γ, ROR-rt, and IL-17A in DNCB-induced mice skin lesions

**FIGURE 6:**
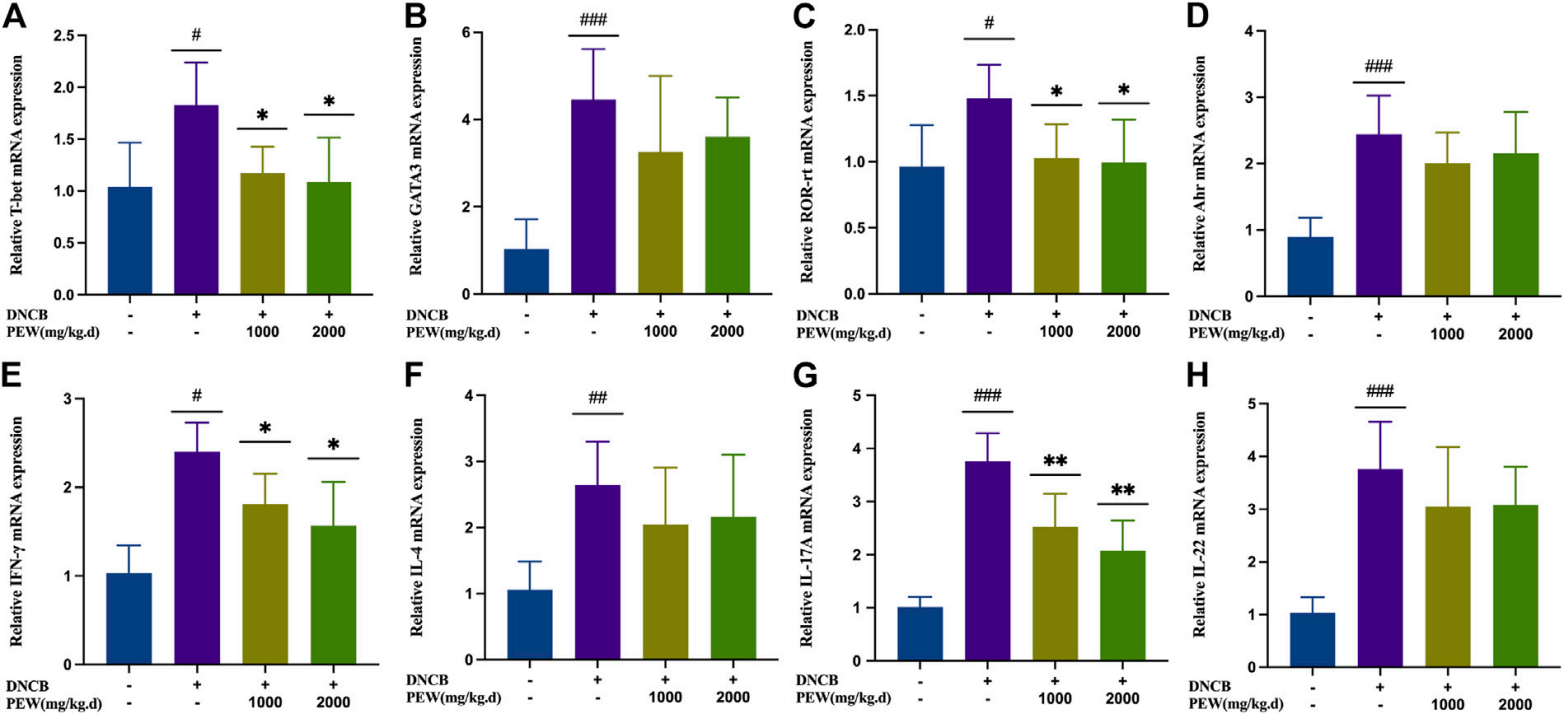
PEW reduces mRNA expression of Th1 and Th17 cytokines and nuclear transcript factors (IFN-γ, T-bet, IL-17, and ROR-rt) in DNCB-induced mice skin lesions. **(A–H)**. Relative mRNA expression levels of different nuclear transcript factors and cytokines (*n* = 5/group): **(A)** T-bet, **(B)** GATA-3, **(C)** ROR-rt, **(D)** AHR, **(E)** IFN-γ, **(F)** IL-4, **(G)** IL-17A, and **(H)** IL-22. The data were presented as means ± SD. # *p* < 0.05, ## *p* < 0.01, ### *p* < 0.001 VS. the normal group; * *p* < 0.05, ** *p* < 0.01 VS. the model control group.

To further confirm the effect of PEW on different CD4^+^ Th cell subsets, mRNA expression of the nuclear transcription factor and cytokine of Th1 (T-bet and IFN-γ), Th2 (GATA-3 and IL-4), Th17 (ROR-rt and IL-17A), and Th22 (Ahr and IL-22) in different groups of mouse skin tissue were measured by RT-qPCR. Consistent with previous results, all nuclear transcription factors and cytokines in the AD model group were significantly increased compared with the normal control group, and the mRNA expression of Th1 (T-bet and IFN-γ) and Th17 (ROR-rt and IL-17A) nuclear transcription factors and cytokines were significantly suppressed in PEW-treated groups compared to the AD model group (Figure 6). Meanwhile, in PEW-treated AD mice groups, the mRNA expression of Th2 (GATA-3 and IL-4) and Th22 (Ahr and IL-22) were not markedly lower than in the untreated mice group (Figure 6).

### 3.5 PEW inhibits the nuclear factor (NF)-κB and mitogen-activated protein kinases (MAPKs) signaling pathways

**FIGURE 7:**
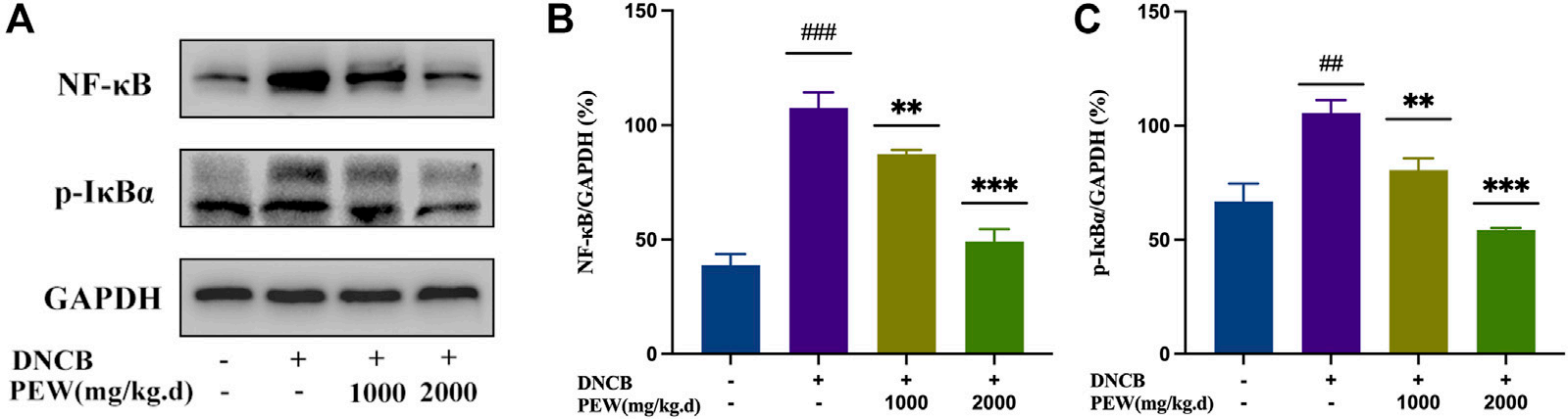
PEW inhibited the NF-κB signaling pathway. **(A)**. Representative images of Western Blotting assays of NF-κB and p-IκBα. **(B)**. Expression of NF-κB relative to GAPDH (*n* = 3/group). **(C)**. Expression of p-IκBα relative to GAPDH (*n* = 3/group). The data were presented as means ± SD. ## *p* < 0.01, ### *p* < 0.001 VS. the normal group; ** *p* < 0.01, *** *p* < 0.001 VS. the model control group.

**FIGURE 8:**
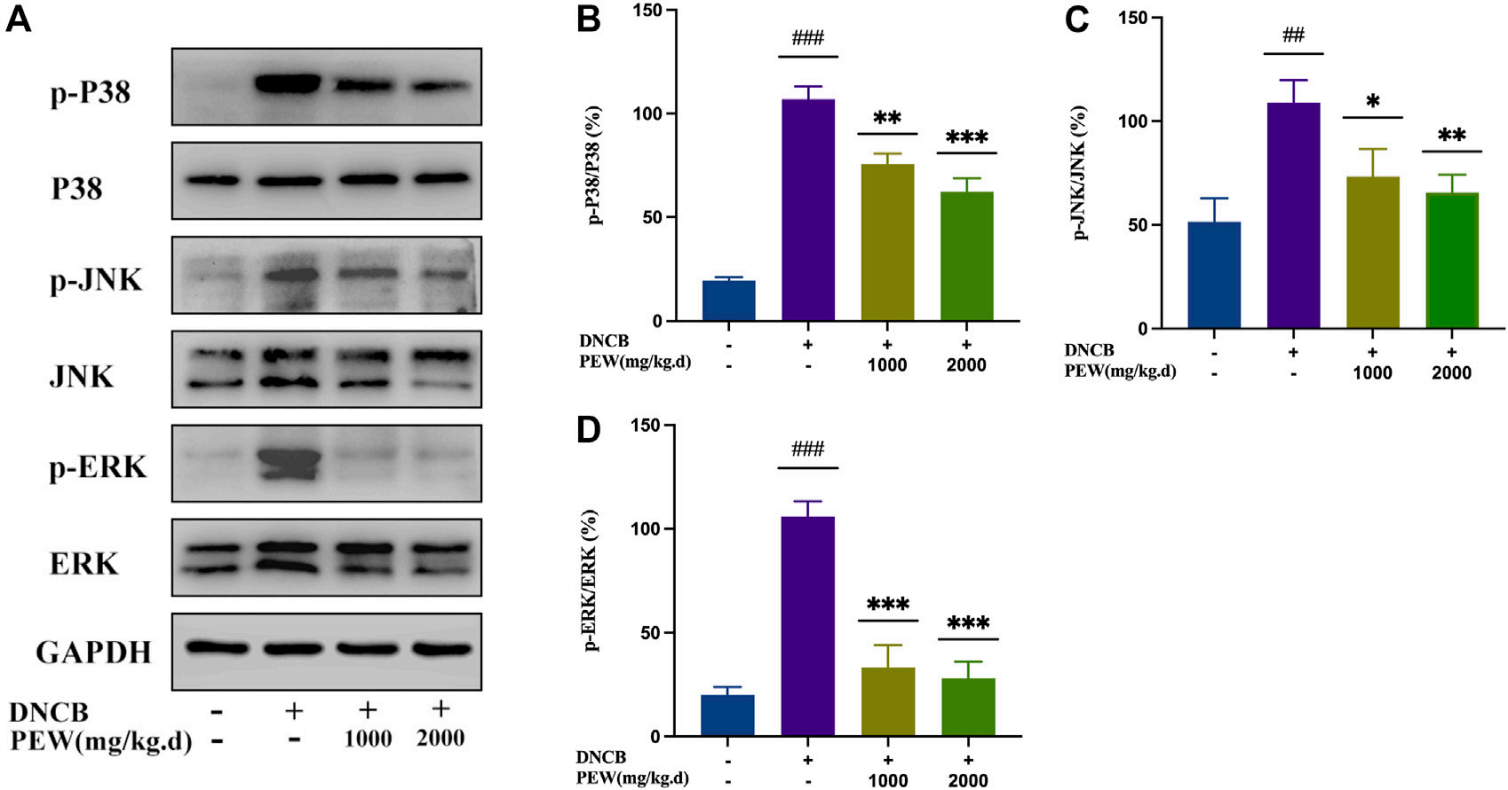
PEW suppressed the phosphorylation level of mitogen-activated protein kinases (MAPKs). **(A)**. Representative images of Western Blotting assays of p-p38, p38, p-JNK, JNK, p-ERK, and ERK. **(B–D)**. Expression of **(B)** p-p38 relative to p38, **(C)** p-JNK relative to JNK, and **(D)** p-ERK relative to ERK (*n* = 3/group). The data were presented as means ± SD. ## *p* < 0.01, ### *p* < 0.001 VS. the normal group; * *p* < 0.05, ** *p* < 0.01, *** *p* < 0.001 VS. the model control group.

The NF-κB signaling pathway and the phosphorylation of MAPKs (p38, ERK, and JNK) pathways were reported to be activated in DNCB-induced AD-like skin lesions in mice. In the present study, the effect of PEW on the activation of NF-κB and the phosphorylation of IκB-α and MAPKs (p38, ERK, and JNK) signaling pathways were measured by Western Blotting analysis with mice dorsal skin tissue. As shown in Figures 7, 8, the level of NF-κB, p-IκBα, p-P38/P38, p-ERK/ERK, and p-JNK/JNK in the DNCB-induced AD model group were significantly increased compared to the normal control group. However, in comparison with the AD model group, the expression of NF-κB and the phosphorylation of IκBα and MAPKs (p38, ERK, and JNK) were significantly suppressed in the PEW-treated groups in a dose-dependent manner.

## 4 Discussion

Wuguchong (maggot) has been widely used in the wound field since antiquity, including diabetic foot ulcers, chronic wounds, pressure ulcers, venous ulcers, etc., due to its pharmacological properties of antibacterial activity, anti-inflammatory activity, proangiogenic function, and neurogenesis (Yan et al., 2018; Moya-López et al., 2020). Besides, some studies have proven the immunomodulatory function of the maggot, which is considered as a protective behavior of maggot therapy (Yan et al., 2018). Although AD skin lesion is apparently different from the classical wounds discussed above, the pathophysiology mechanism of AD is well known for immune dysregulation and inflammatory responses (Langan et al., 2020). Moreover, recent research data demonstrate that polysaccharides extracted from some natural products have a positive effect in treating AD-like skin lesions in mice and humans (Wu et al., 2011; Nam et al., 2013; Lin et al., 2017; Ashigai et al., 2018; Motoyama et al., 2018; Tian et al., 2019). In previous studies, the polysaccharide extracted from Wuguchong (PEW) was found as a bioactive ingredient to prevent obesity and relieve intestinal inflammation caused by drug side effects, partly through the modulation of inflammatory responses (Wang et al., 2020; Shi et al., 2022). However, the effect of PEW on AD has not been investigated so far. In this study, we interrogated the therapeutic effects of PEW on DNCB-induced AD-like skin lesions in Balb/c mice and examined its immunoregulation effects on CD4^+^ Th cell subsets. The results demonstrated that PEW markedly alleviates DNCB-induced AD-like symptoms mainly *via* inhibition of Th1 and Th17 responses and downregulation of NF-κB and MAPK signaling pathways.

Essential features of AD are intense itching and eczematous lesions, with a relapsing or chronic disease course (Langan et al., 2020). Histologically, “acute” papular skin lesions are characterized by spongiosis (profound intercellular edema in the epidermis), whereas “chronic” lichenified lesions are characterized by a hyperplastic epidermis with prominent hyperkeratosis, and elongation of the rete ridges, but minimal spongiosis (Bieber, 2010). In the current study, the epidermal and dermal thicknesses were significantly increased in DNCB-induced AD mice, and oral administration of PEW effectively reversed the trends [Figures 3A(a), B, C]. Increased infiltration of mast cells is another histological feature of AD (Bieber, 2010). Intensive degranulation of mast cells is often observed in the inflammatory skin region in AD and is proven to be related to the severity of the disease (Irani et al., 1989; Zhao et al., 2006; Liu et al., 2011). Mast cells are sensitized by IgE through the high-affinity receptor of IgE (FcεRI) on their cell surface, then involved in the production of some cytokines, chemokines, and growth factors, thus regulating the recruitment, trafficking, and functions of cells related to the skin inflammatory response (Liu et al., 2011). Polyvalent antigens recognized by bound IgE aggregate FcεRI to activate mast cells, allowing for the initiation of an immediate hypersensitivity response (or early-phase), and also the late-phase response, which is central to the pathogenesis of allergic diseases (Stone et al., 2010; Galli and Tsai, 2012). Immediate response determined by rapidly synthesized lipid mediators and prefabricated mediators leads to skin erythema, edema, and itching (Stone et al., 2010). Histamine, the key preformed mediator produced by mast cells, plays a crucial role in the development of allergic-inflammatory responses by promoting the release of some chemokines and pro-inflammatory cytokines like IL-8, RANTES, IL-6, IL-1α, and IL-1β (Jutel et al., 2009). At the same time, histamine is an important regulator of epithelial and endothelial cell barrier function, which directly changes vascular permeability and leads to the infiltration of leukocytes and the formation of edema (Jutel et al., 2009). In this study, PEW reduces the infiltration of mast cells in the skin of DNCB-induced AD mice and downregulates the levels of serum IgE and histamine [Figures 3A(b), D; Figure 4], suggesting that PEW ameliorates AD-like symptoms in mice partly by affecting the IgE-mast-cell axis. PEW decreases the IgE level and then inhibits the activation of mast cells due to decreased combination of receptor FcεRI with IgE, accompanied by a reduction in histamine secretion, thus relieving allergic-inflammatory responses.

Cutaneous inflammation is a hallmark of AD, which is characterized by the infiltration of inflammatory cells in sequential and progressive patterns, particularly by CD4^+^ cells (Weidinger and Novak, 2016). Specifically, both Th2 and Th22 activation are hallmarks of AD, with some Th1 and Th17 components (Mansouri and Guttman-Yassky, 2015). Th2 and Th22 activation induce the acute phase of AD and show a progressive intensification to the chronic phase (Gittler et al., 2012), while IL-17 sees a decrease in the progression (Souwer et al., 2010), lower levels of IL-17 during the chronic phase of AD indicate a possible Th17-to-Th1 shift in later phases of the disease (Cesare et al., 2008; Koga et al., 2008). Known as a biphasic inflammation, although the Th2-predominant immune response is closely associated with the acute onset of the disease, there is a Th2-to-Th1 shift in the progression of AD, in which the chronic lesion is predominated with a Th1-biased response (Tian et al., 2019); Th17 activation also seems to play a role in a prolonged exaggeration of AD lesions and are correlated with the severity of the disease (Koga et al., 2008). Even though Th17 cells are not closely associated with Th1/Th2 balance, there is a significant correlation between the percentage of Th17 cells and IFN-γ-producing Th1 cells, but not IL-4-producing Th2 cells, suggesting Th17 cells work as an enhancer in the development of AD, but not an immune-polarizer (Koga et al., 2008). In the current study, the treatment of PEW showed significant potency in decreasing the frequency of Th1 and Th17 cells (Figure 5), as well as suppressing the mRNA expressions of Th1 and Th17 cytokines and nuclear transcription factors in AD-lesion inflammation (Figure 6), which indicates that PEW mainly modulates the pathogenesis of chronic AD skin inflammation. In chronic AD, Th1 cells and Th1-related cytokine IFN-γ contribute to dermal thickening in the AD mouse model (Spergel et al., 1999), so depressed Th1 activation by PEW leads to a significant decrease in the dermal thickness of AD mice [Figures 3A(a), C]. Th17 cells are characterized by the secretion of inflammatory cytokines like IL-17A and IL-17F (Cesare et al., 2008). IL-17A exerts a pro-allergic action on B cells by promoting IgE production (Milovanovic et al., 2010). The reduced IL-17A production (Figure 6G) contributes to decreasing the IgE level (Figure 4A), thus affecting the IgE-Mast-cell axis and relieving the related allergic-inflammatory responses in mice with AD. Besides, IL-17A induces p50 and p65 NF-κB activations, and NF-κB is the most important downstream target of IL-17A (Huang et al., 2007; Milovanovic et al., 2010). IL-17A triggers rapid degradation of IκBα and subsequent translocation of NF-κB into the B-cell nucleus, followed by the p50 and p65 nuclear translocation, and significant increases in their DNA-binding activities to the consensus sequences of NF-κB (Huang et al., 2007; Milovanovic et al., 2010). So, a lower IL-17A level (Figure 6G) caused by PEW plays a role in suppressing the degradation of IκBα and subsequent activation of NF-κB (Figure 7).

On the other hand, NF-κB is involved in the regulation of T-cell differentiation and effector function. Canonical NF-κB mediates the differentiation of CD4^+^ T cells *via* regulating cytokine production in innate immune cells and T-cell intrinsic mechanisms (Oh and Ghosh, 2013; Liu et al., 2017). NF-κB promotes Th1 cell differentiation, substantial IFN-γ production, and efficient clonal expansion, independent of its role in APC production of IL-12 (Corn et al., 2003). Diminished NF-κB activation in T cells can lead to preferential impairment of Th1 responses, as evidenced by significant inhibition of delayed-type hypersensitivity responses, and IFN-γ production (Aronica et al., 1999). Several NF-κB members are involved in promoting Th17 responses: such as NFKB1, the IκB-like molecule p105, T-cell-specific IKKβ, c-Rel and RelA (Liu et al., 2017), in particular, c-Rel and RelA, which directly bind to the promoter region and upregulate the mRNA expression of Th17 nuclear transcription factor ROR-γt and induce Th17 cell generation (Ruan et al., 2011; Oh and Ghosh, 2013). As indicated above, the NF-κB pathway is crucial in mediating the generation of Th1 and Th17 cells. In the present study, PEW significantly reduced the phosphorylation level of IκBα (Figures 7A, C) and inhibited the activation of the NF-κB signal pathway (Figures 7A, B), which in turn contributes to reducing the generation of Th1 and Th17 cells and suppressing Th1 and Th17 responses (Figures 5, 6).

Mitogen-activated protein kinases (MAPKs), including the p38, ERK, and JNK subfamilies, are the other classic inflammation-related pathways. Cross-talking with NF-κB signaling, activation of MAPKs contributes to the induction of several genes which together mediate the inflammatory response (Arthur and Ley, 2013). Phosphorylation of MAPKs leads to inflammatory mediators’ production and promotes the allergic inflammatory response (Barnes, 2011; Arthur and Ley, 2013). Among the mitogen-activated protein kinases, p38 and ERK MAPK pathways are involved in the pathogenesis of inflammatory skin diseases including AD (Johansen et al., 2005; Lee et al., 2022; Zeze et al., 2022), and the JNK pathway is also activated in atopic dermatitis (Lu et al., 2018). Increasing evidence has demonstrated the efficacy of p38 and ERK MAPK inhibitors on AD, which may work as potential therapeutic targets (Lee et al., 2022; Zeze et al., 2022). In the current study, PEW significantly inhibits the activation of p38, ERK, and JNK MAPKs (Figure 8), not only contributing to the downregulation of the NF-κB signaling pathway but also blocking the production of inflammatory mediators, thus relieving skin inflammation in AD mice. Additionally, p38 MAPK regulates the differentiation of naive CD4 T cells (Dodeller et al., 2005) and mediates the function of Th1 and Th17 cells and the production of their related cytokines (Rincón et al., 1998; Noubade et al., 2011), indicating that the inactivation of p38 MAPK by PEW (Figures 8A, B) may play a role in inhibiting Th1 and Th17 responses in AD (Figures 5, 6).

Furthermore, recent evidence indicates that obesity can also influence the immune system and is associated with atopic dermatitis (Zhang and Silverberg, 2015; Ali et al., 2018; Bapat et al., 2022). A high-fat diet (HFD) markedly increased inflammatory response in obese mice with AD compared to lean mice, and obesity increased disease severity in AD mouse models by converting the classical Th2—predominated AD to a more severe disease with prominent Th17 inflammation (Bapat et al., 2022). A western diet (WD), characterized by rich fat and high sugar enhances susceptibility to dermatitis, a long-term WD intake leads to dermatitis with Th2 and Th17 pathway features in mice (Jena et al., 2019), while a short-term WD induced a Th1/Th17 response biased skin inflammation (Shi et al., 2020); dietary components, rather than obesity, can cause mild, even clinically significant skin inflammation (Shi et al., 2020). In addition, numerous studies have shown a gut microbial dysbiosis association in AD patients, the gut microbiome may contribute to the development, course, and severity of AD *via* immunologic, metabolic, and neuroendocrine pathways (Lee et al., 2018; De Pessemier et al., 2021; Moniaga et al., 2022). Neonatal gut microbiome dysbiosis is associated with childhood atopy and promotes CD4^+^ T cell dysfunction (Fujimura et al., 2016). A recent study demonstrated polysaccharides of Tremella fuciformis alleviate AD in mice by regulating immune response and gut microbiota (Xie et al., 2022). In our previous study, PEW reduces high-fat diet-induced obesity in mice *via* the regulation of gut microbiota (Wang et al., 2020), we hypothesize the gut microbiome regulation function of PEW may play a role in alleviating skin inflammation of AD and influencing CD4^+^ T cell differentiation, also, it is possible that PEW ameliorates AD *via* modulating effects of diets. PEW may play its role in AD by influencing the interaction between diets, gut microbes, and immunity. Further studies are warranted to clarify whether PEW can alleviate AD inflammation by regulating intestinal microbiota, mediating diets’ influence, or preventing obesity.

Our current study indicates that PEW significantly inhibits Th1 and Th17 responses in DNCB-induced AD mice, which may be used as a promising immune therapy targeting Th1 and Th17 pathways. However, AD immunoendotypes are in part associated with race or ethnic group (Suaini et al., 2021). For instance, activation of the Th1 and Th17 pathways are absent in Black AD patients; Asian patients have been reported with the activation of Th2 and Th17 pathways, whereas there is mostly activation of the Th2 pathway in European patients (Brunner and Guttman-Yassky, 2019; Ständer, 2021). Besides, the Th17 pathway is activated in certain subtypes such as pediatric, intrinsic, and Asian-origin AD (Yu et al., 2019). Th17 activation in pediatric AD is higher than in adult AD at disease initiation (Esaki et al., 2016); intrinsic AD patients have higher Th17 immune activation than extrinsic ones (Suárez-Fariñas et al., 2013); in European American (EA) AD patients, although Th17 response sees an increased level in intrinsic AD cases, it is downregulated in adults with extrinsic AD, and Asian patients with extrinsic AD have significantly higher Th17 response compared to EA patients with extrinsic AD (Noda et al., 2015; Chan et al., 2018). The efficacy of immune therapy varies in different racial subgroups (Alexis et al., 2019). Although Th2-targeted therapy—the IL-4 receptor antibody dupilumab that blocks both IL-4 and IL-13 signaling has achieved tremendous progress in specific populations (Langan et al., 2020), it clears only approximately one-third of patients, so, additional targeted therapies are required to resolve all subtypes of AD (Ungar et al., 2021). Thereby, PEW is promising to be developed as an immune therapy that targets Th1 and Th17 immune pathways. However, as discussed, the efficacy of PEW in AD may differ among different ethnic groups and subtypes due to the different response profiles to immune therapies. Thus, further studies of PEW in different regions and racial subgroups are warranted to design personalized immune therapy.

## 5 Conclusion

In conclusion, our findings indicate that PEW treatment can alleviate skin inflammation in mice with DNCB-induced AD. PEW significantly inhibited the generation of Th1 and Th17 cells, and profoundly reduced the expression of Th1 and Th17 cytokines and nuclear transcription factors including IFN-γ, T-bet, IL-17, and ROR-rt. Furthermore, we demonstrated the underlying mechanisms of action involved in suppressing inflammation as well as the Th1 and Th17 responses are through the inactivation of NF-κB and MAPK pathways. The potential mechanisms related to regulating gut microbiota and preventing obesity remain to be explored. This study verifies the therapeutic effect of PEW on AD and provides a scientific rationale for this bioactive ingredient as a promising immune therapy for AD. We will purify PEW for further studies in the future.

## Data Availability

The original contributions presented in the study are included in the article/Supplementary Materials, further inquiries can be directed to the corresponding authors.
