# Supplementary figures and images for: Anti-inflammatory and immunomodulatory effects of polysaccharide extracted from Wuguchong (maggot) on 2,4-dinitrochlorobenzene-induced atopic dermatitis in mice
Source: Front Pharmacol. 2023 Mar 22;14:1119103. doi: 10.3389/fphar.2023.1119103 (PMC10073476; doi:10.3389/fphar.2023.1119103)

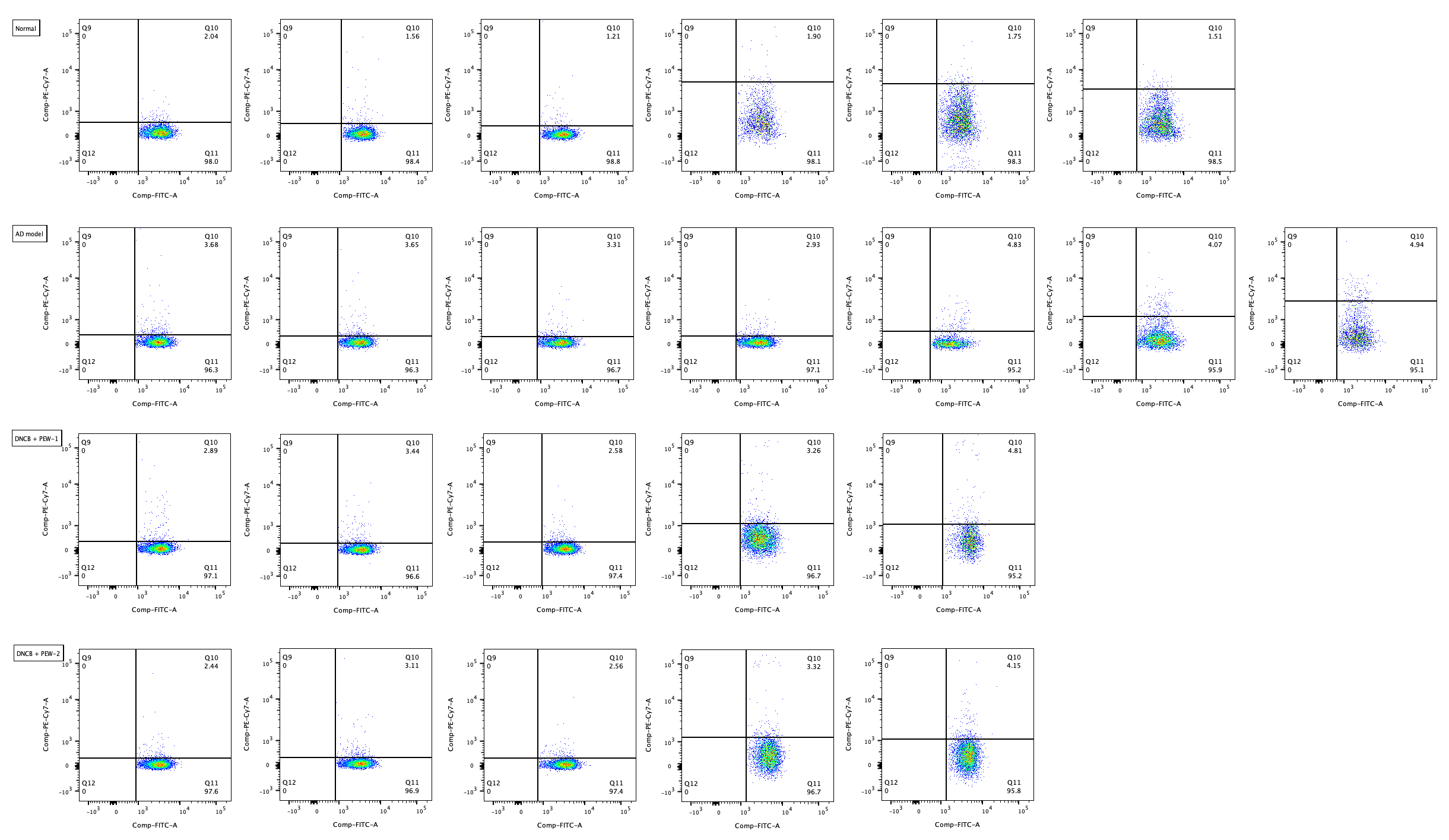

Supplement: Supplementary file 1 [file Image3.TIFF]

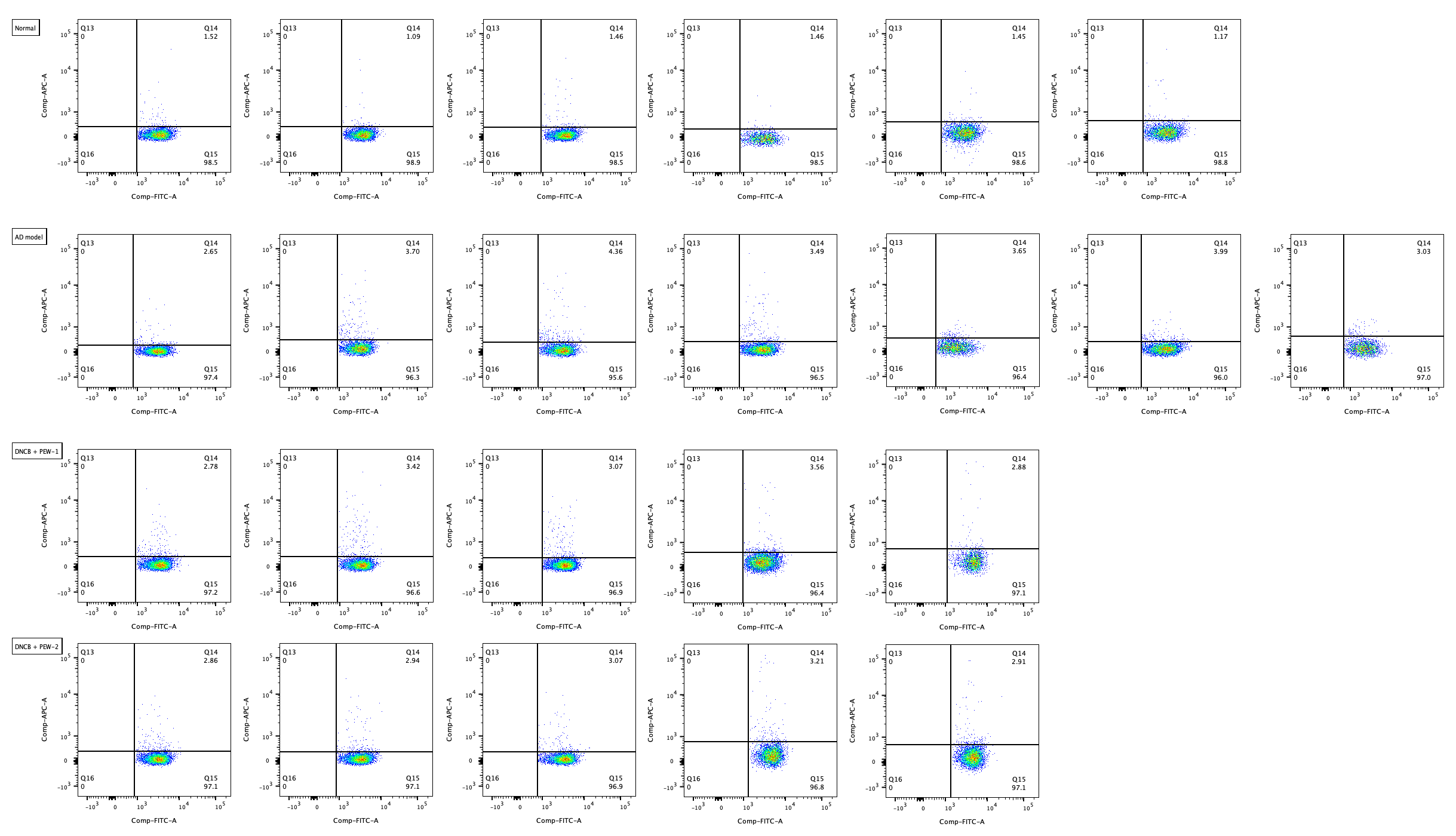

Supplement: Supplementary file 3 [file Image1.TIFF]

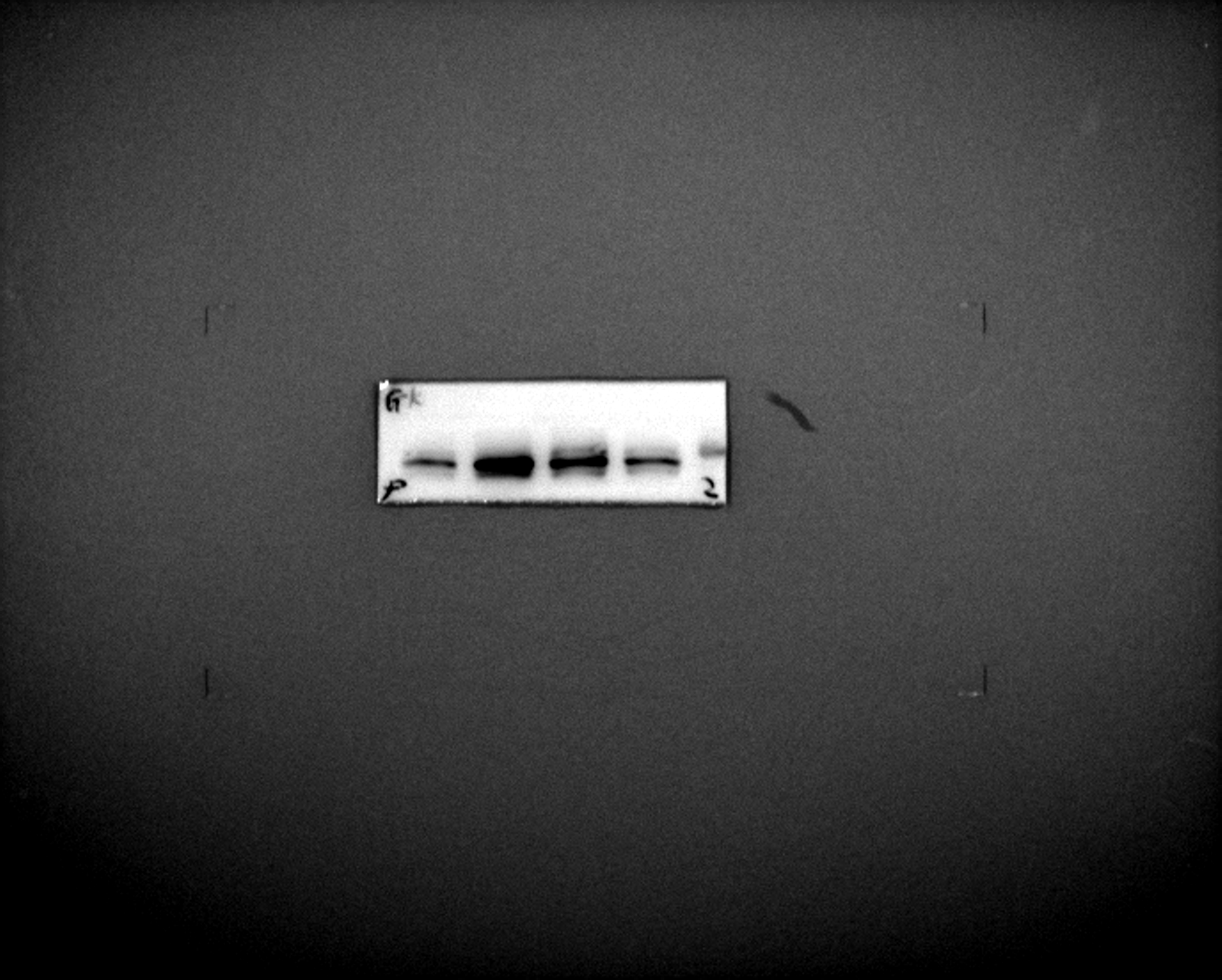

Supplement: Supplementary file 5 [file Image6.TIF]

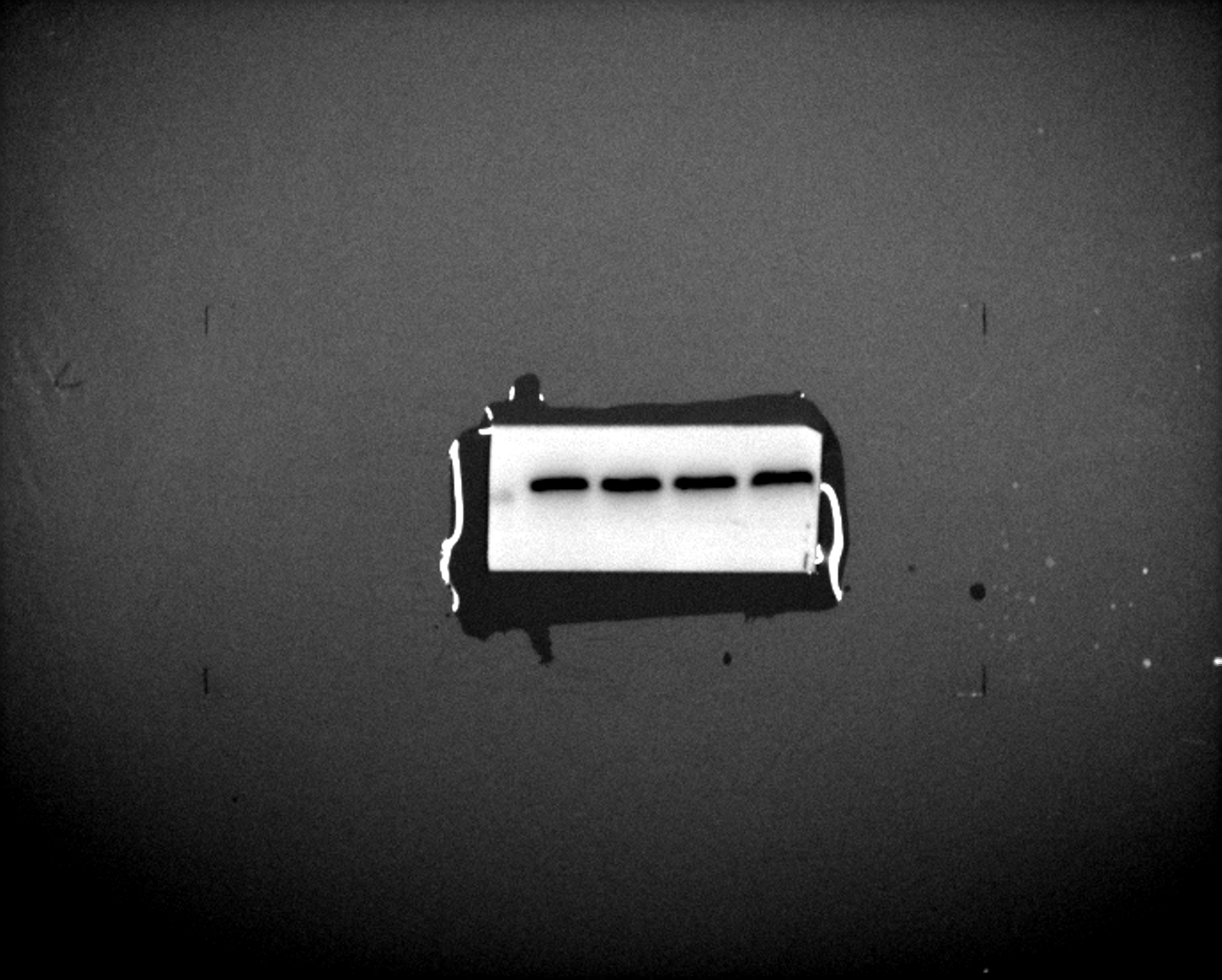

Supplement: Supplementary file 6 [file Image14.TIF]

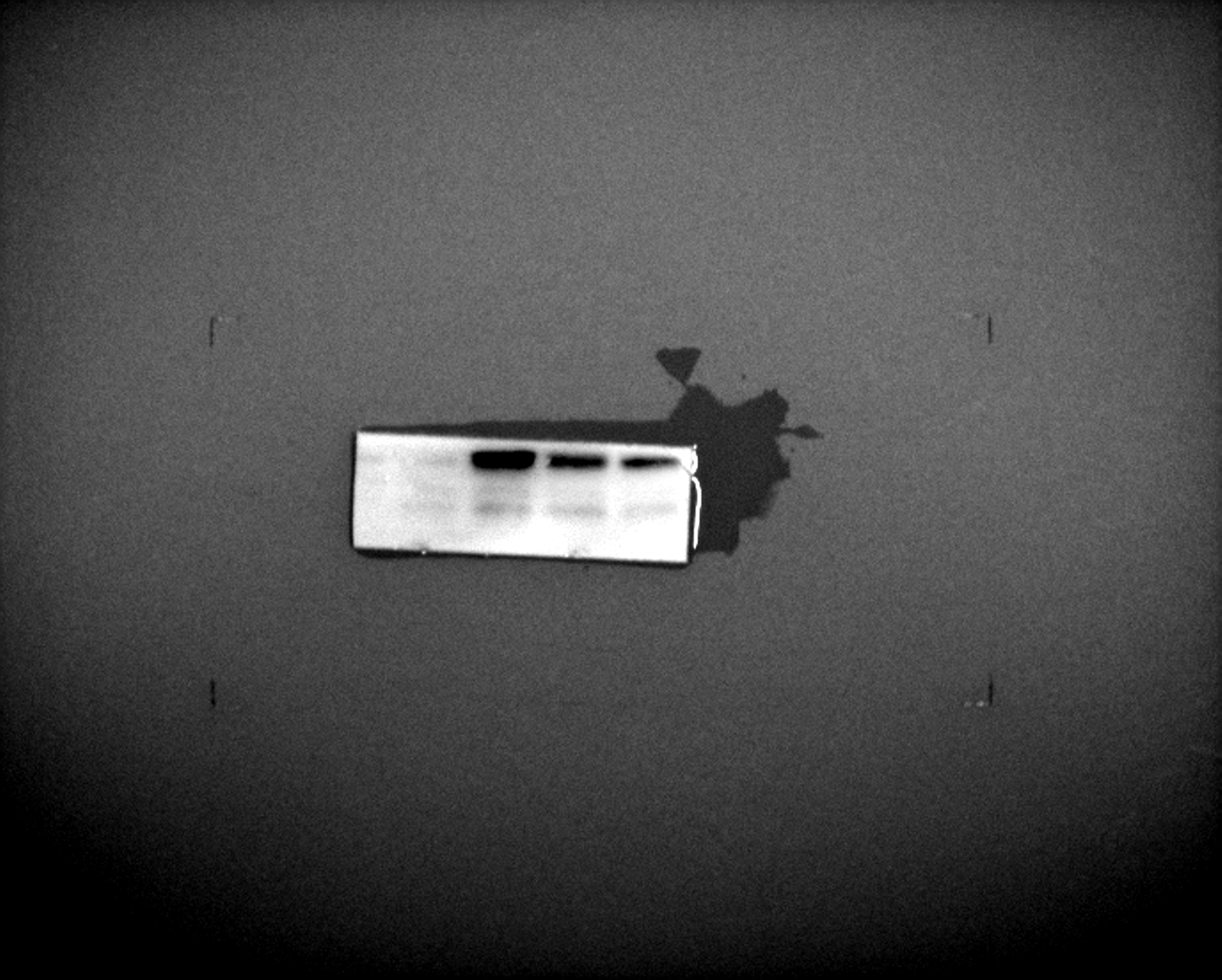

Supplement: Supplementary file 8 [file Image9.TIF]

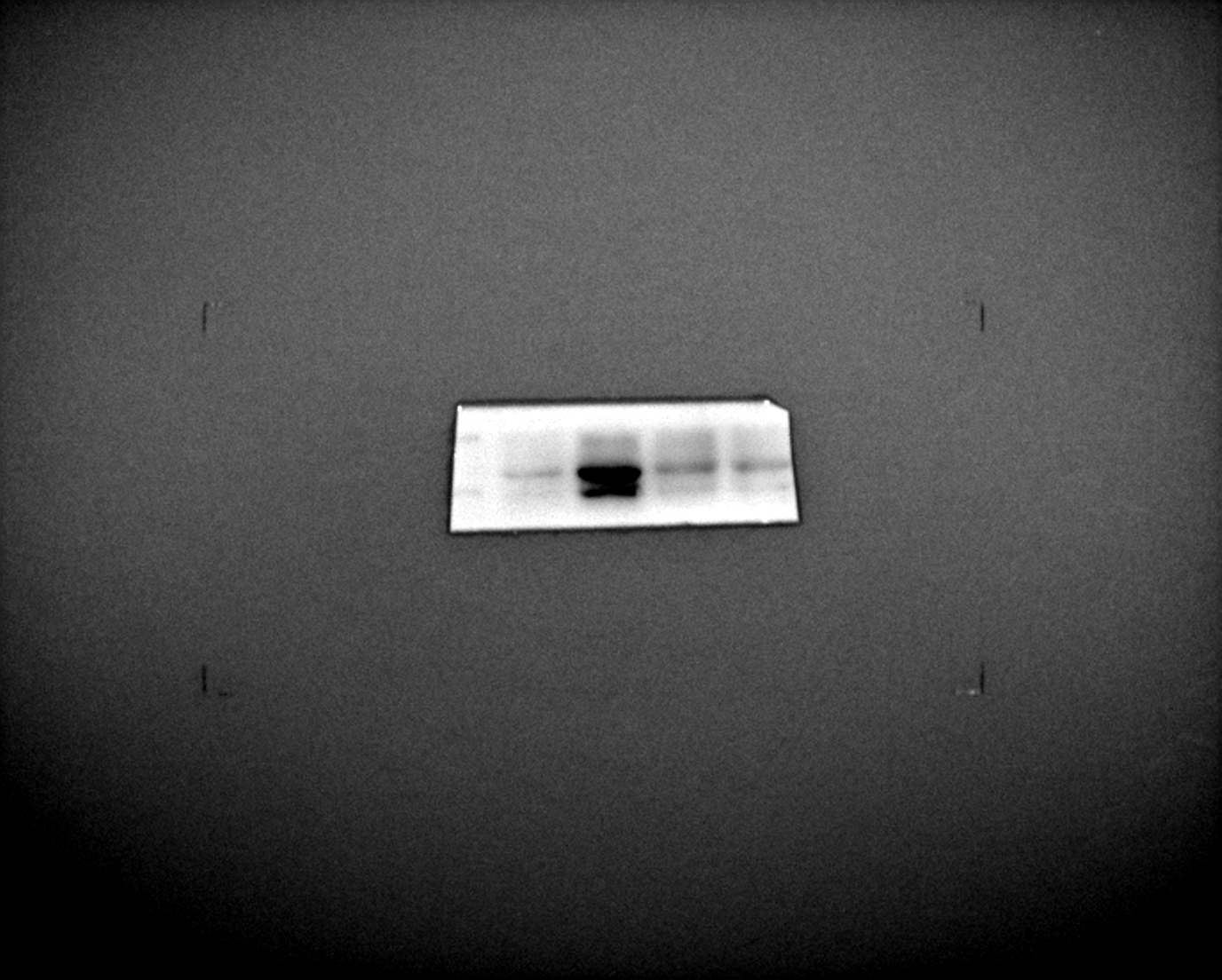

Supplement: Supplementary file 9 [file Image13.TIF]

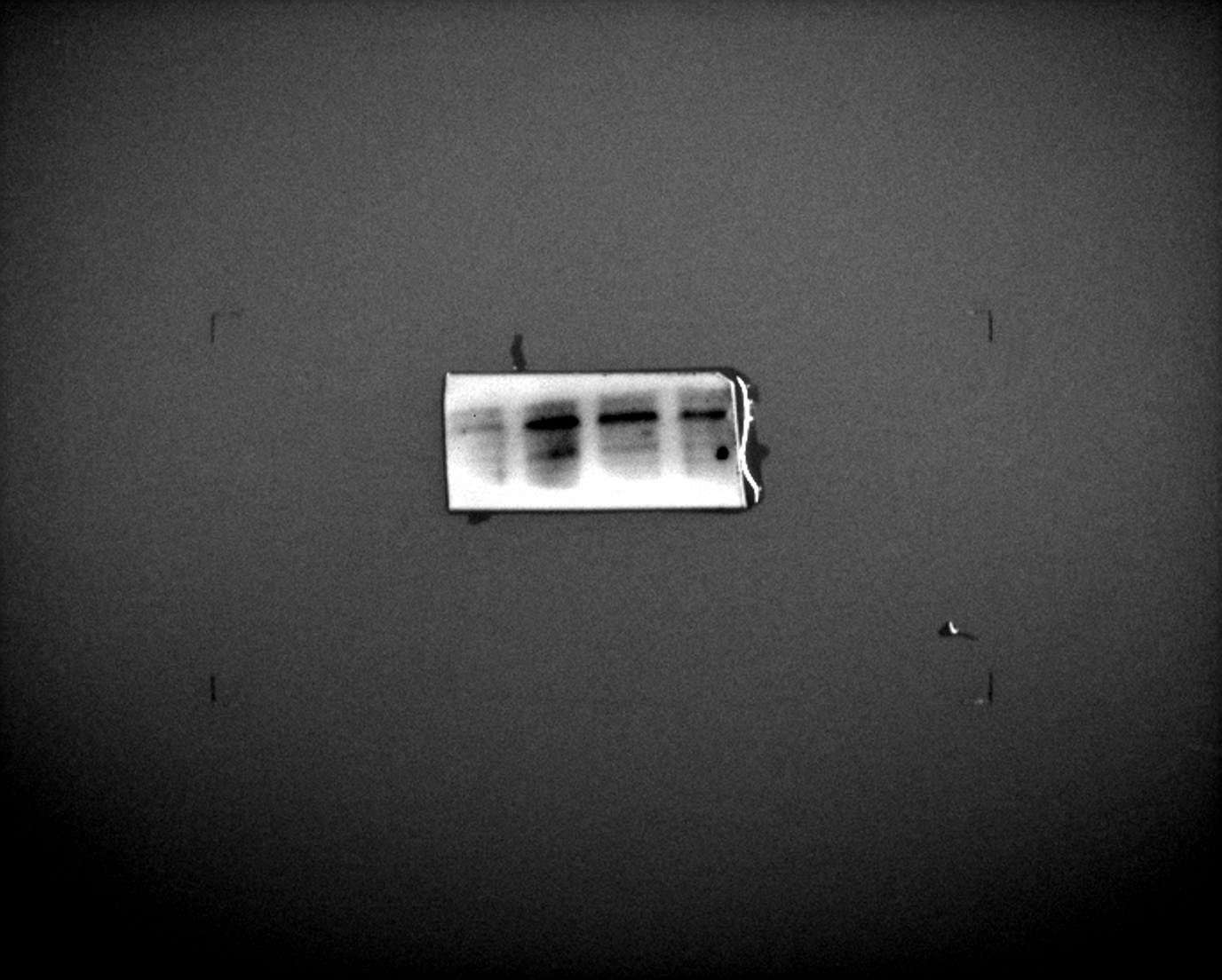

Supplement: Supplementary file 10 [file Image11.TIF]

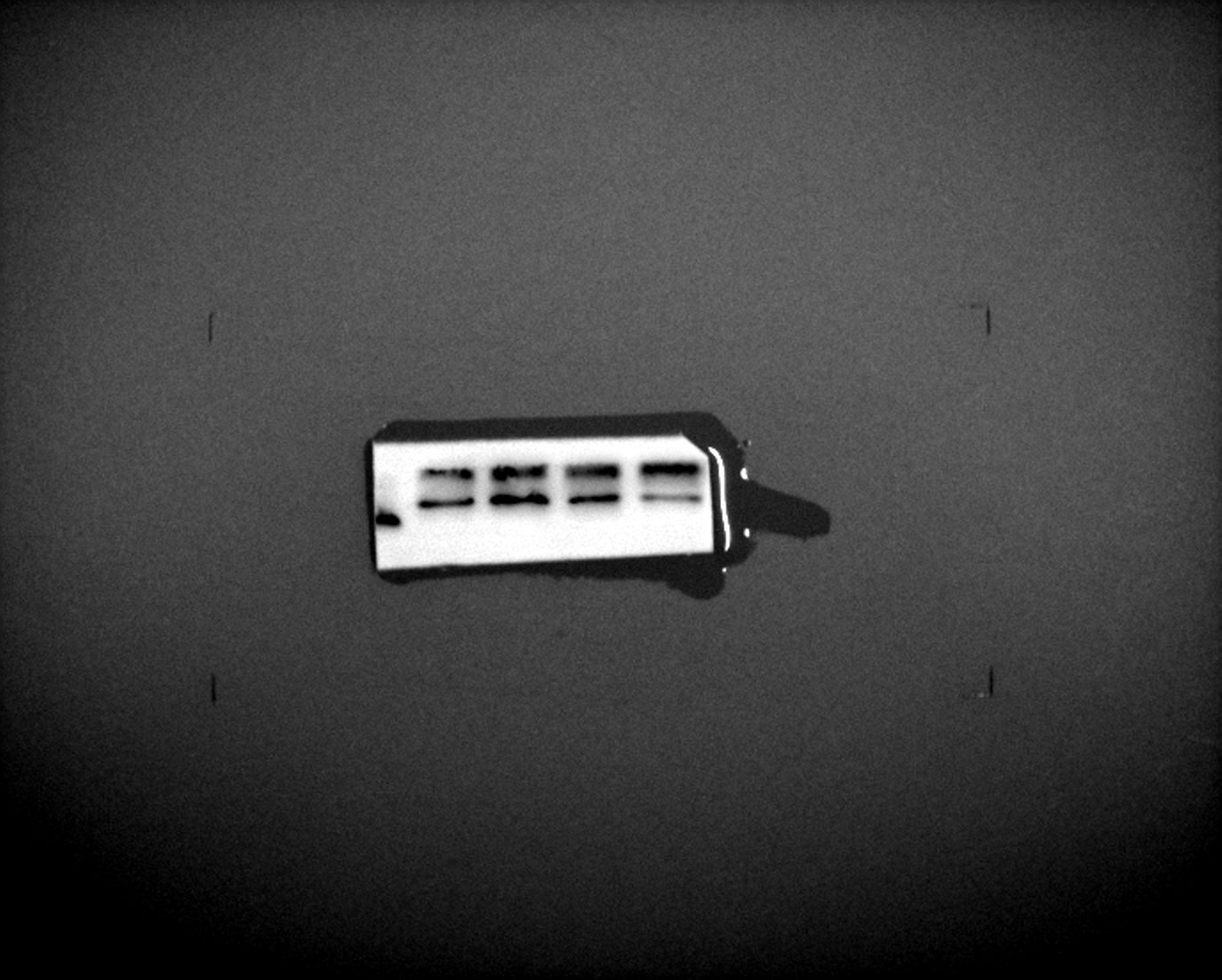

Supplement: Supplementary file 11 [file Image10.TIF]

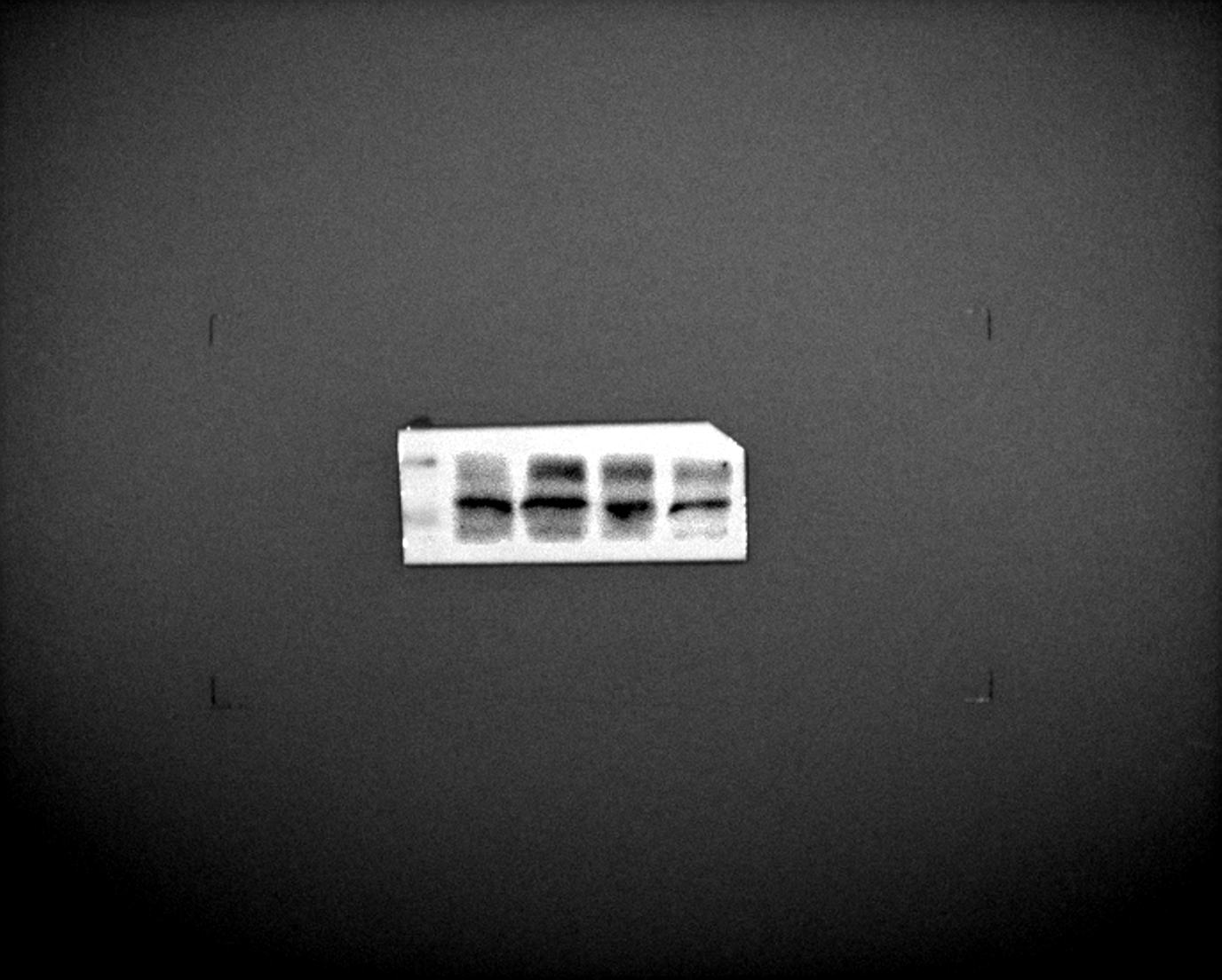

Supplement: Supplementary file 12 [file Image7.TIF]

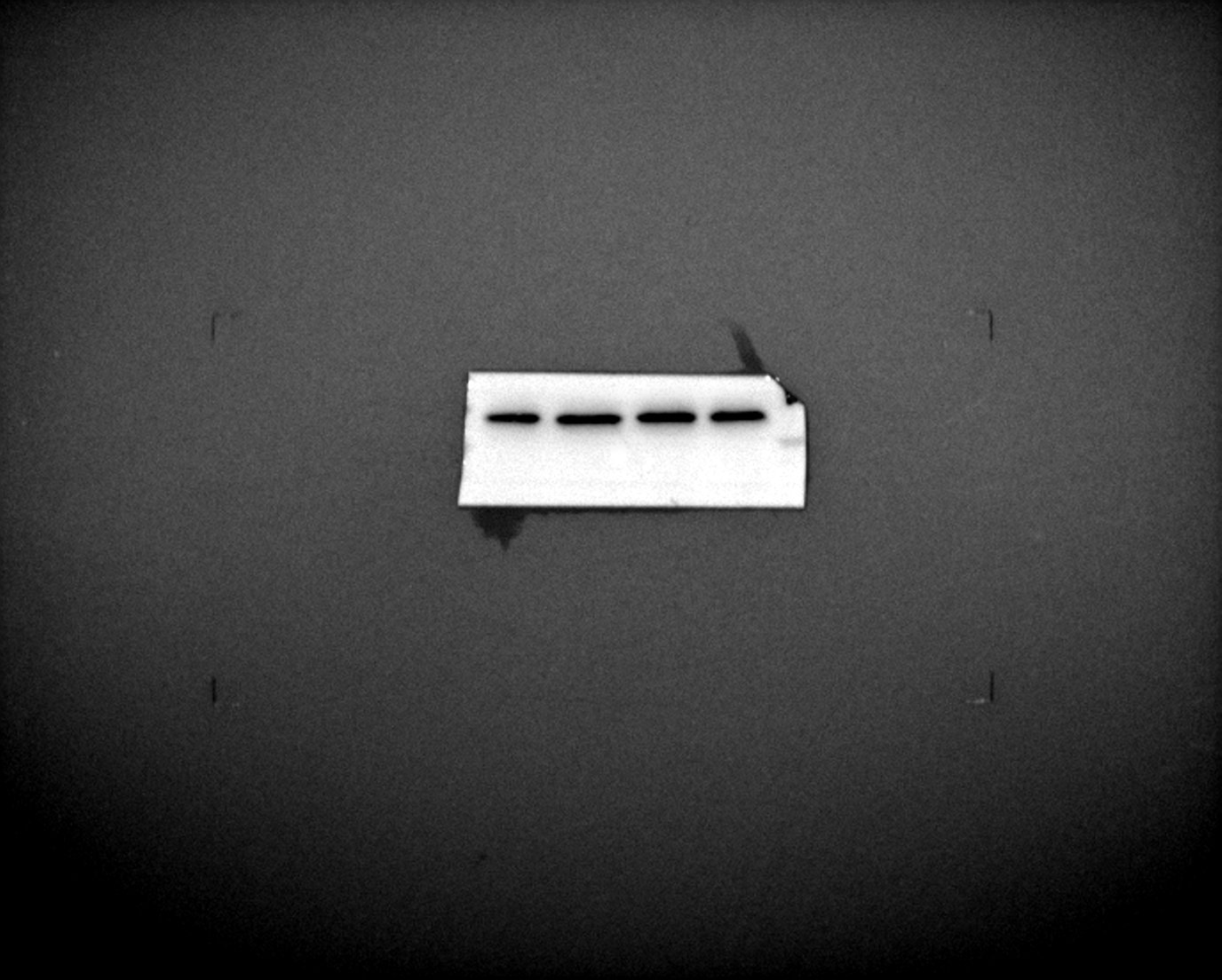

Supplement: Supplementary file 17 [file Image8.TIF]

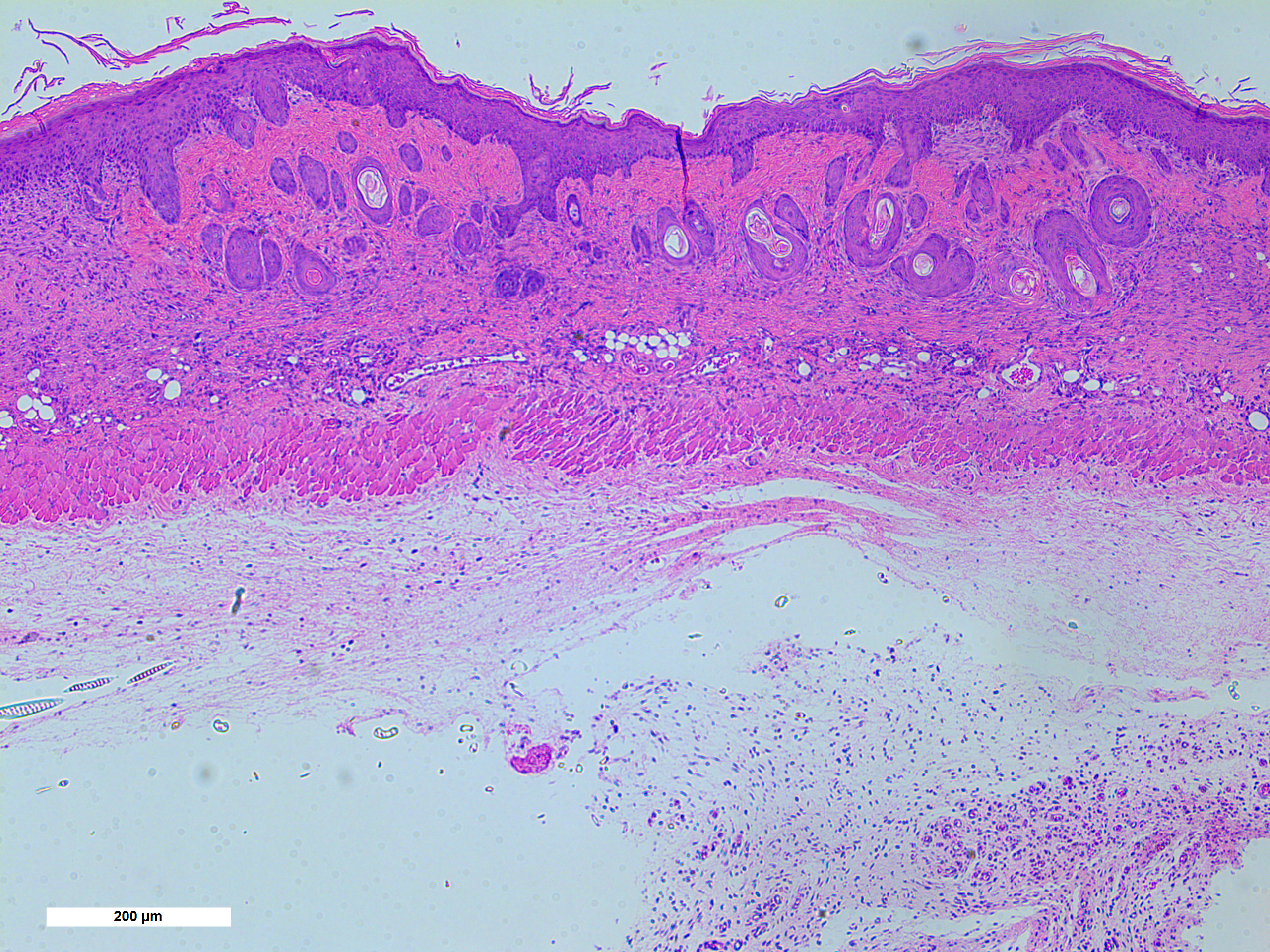

Supplement: Supplementary file 19 [file Image5.TIF]

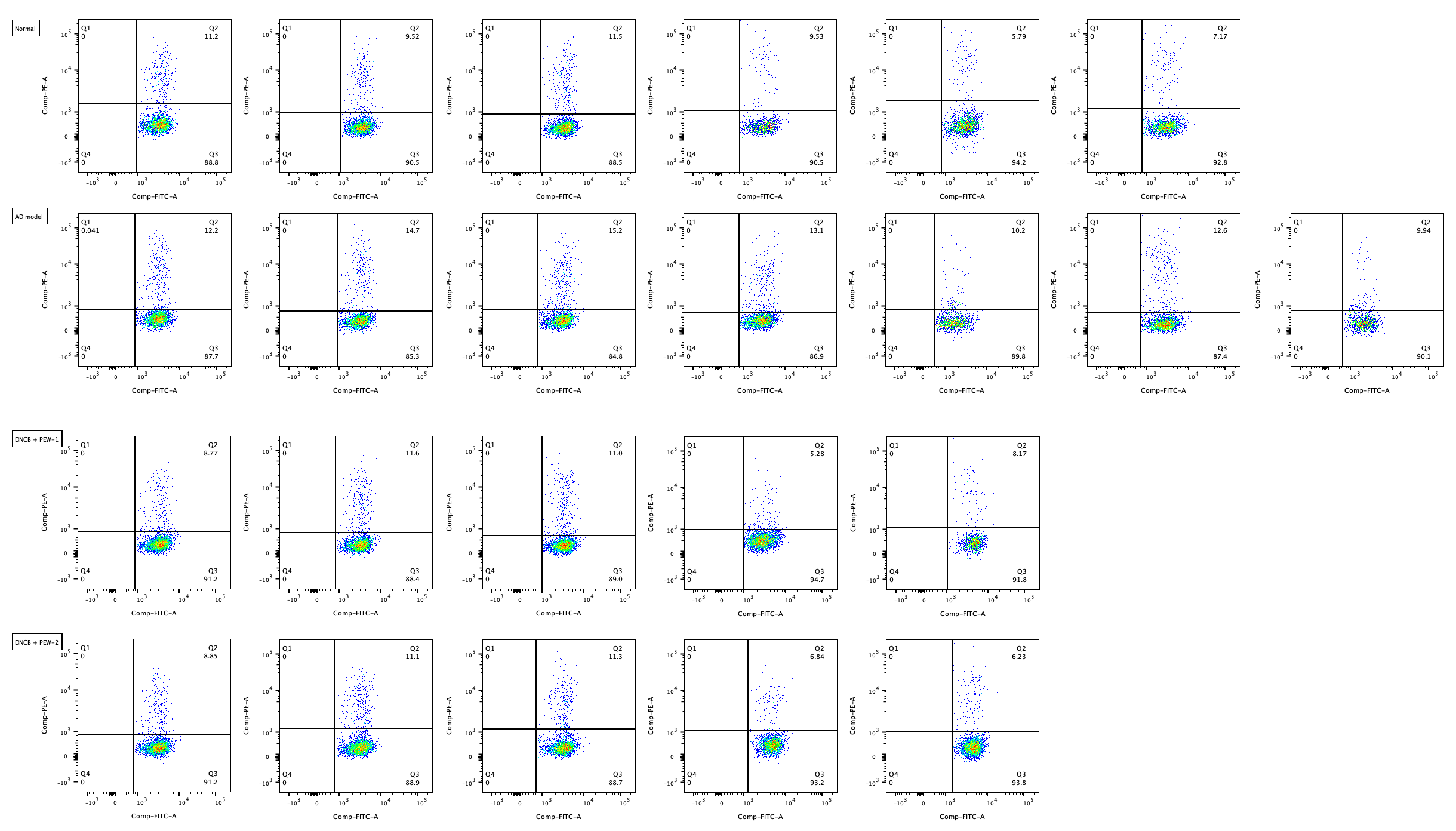

Supplement: Supplementary file 21 [file Image2.TIFF]

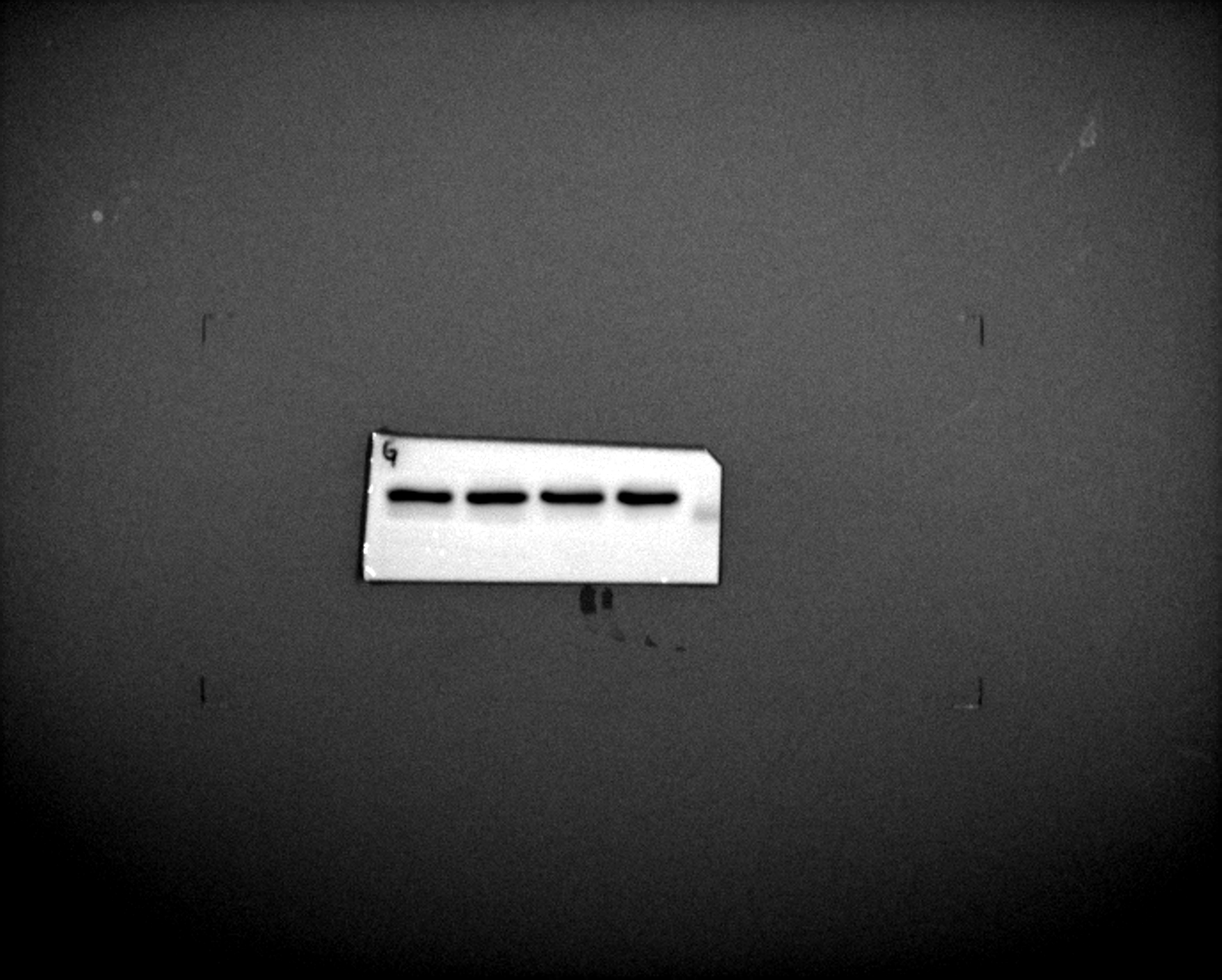

Supplement: Supplementary file 22 [file Image15.TIF]

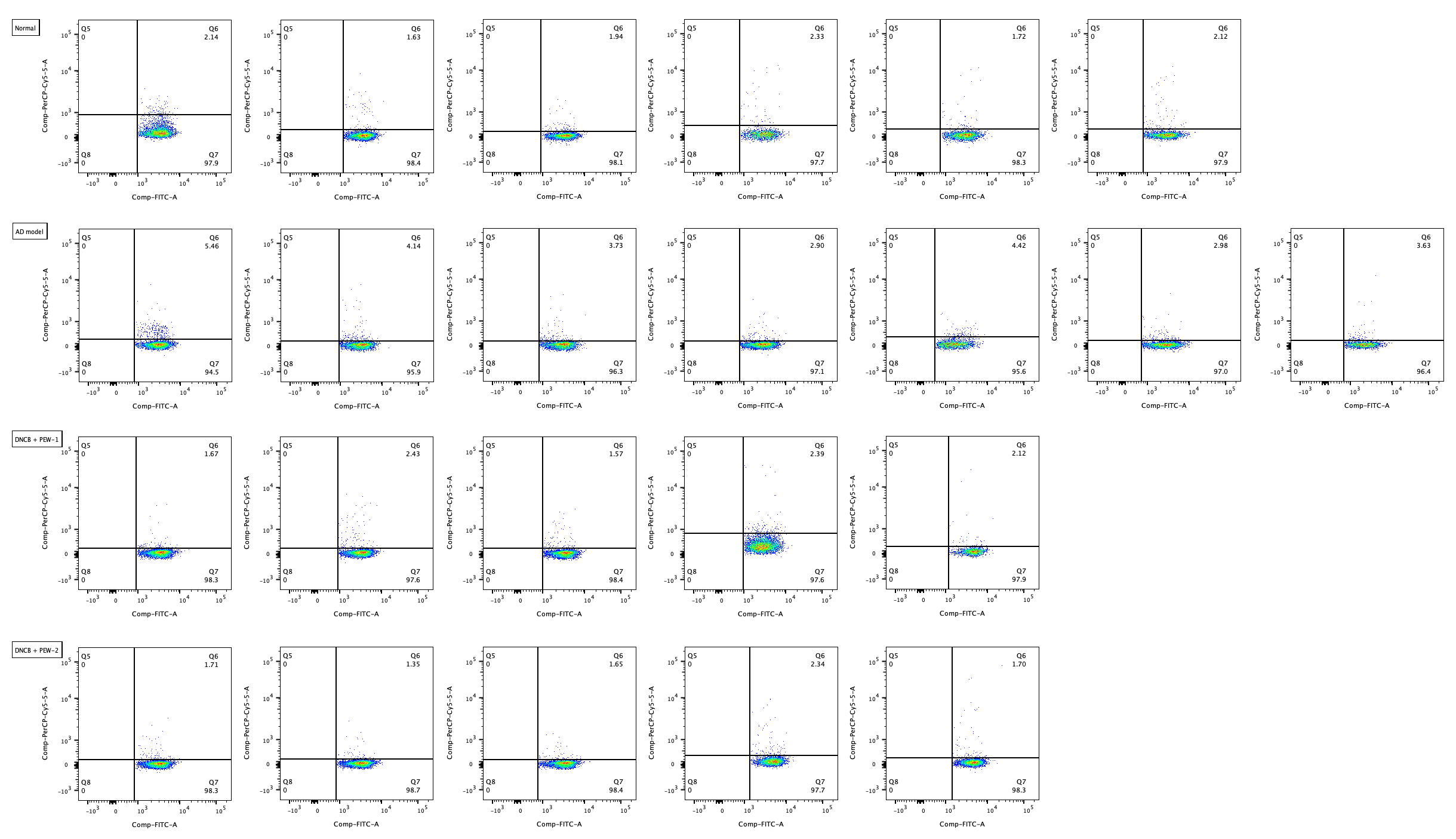

Supplement: Supplementary file 23 [file Image4.TIFF]

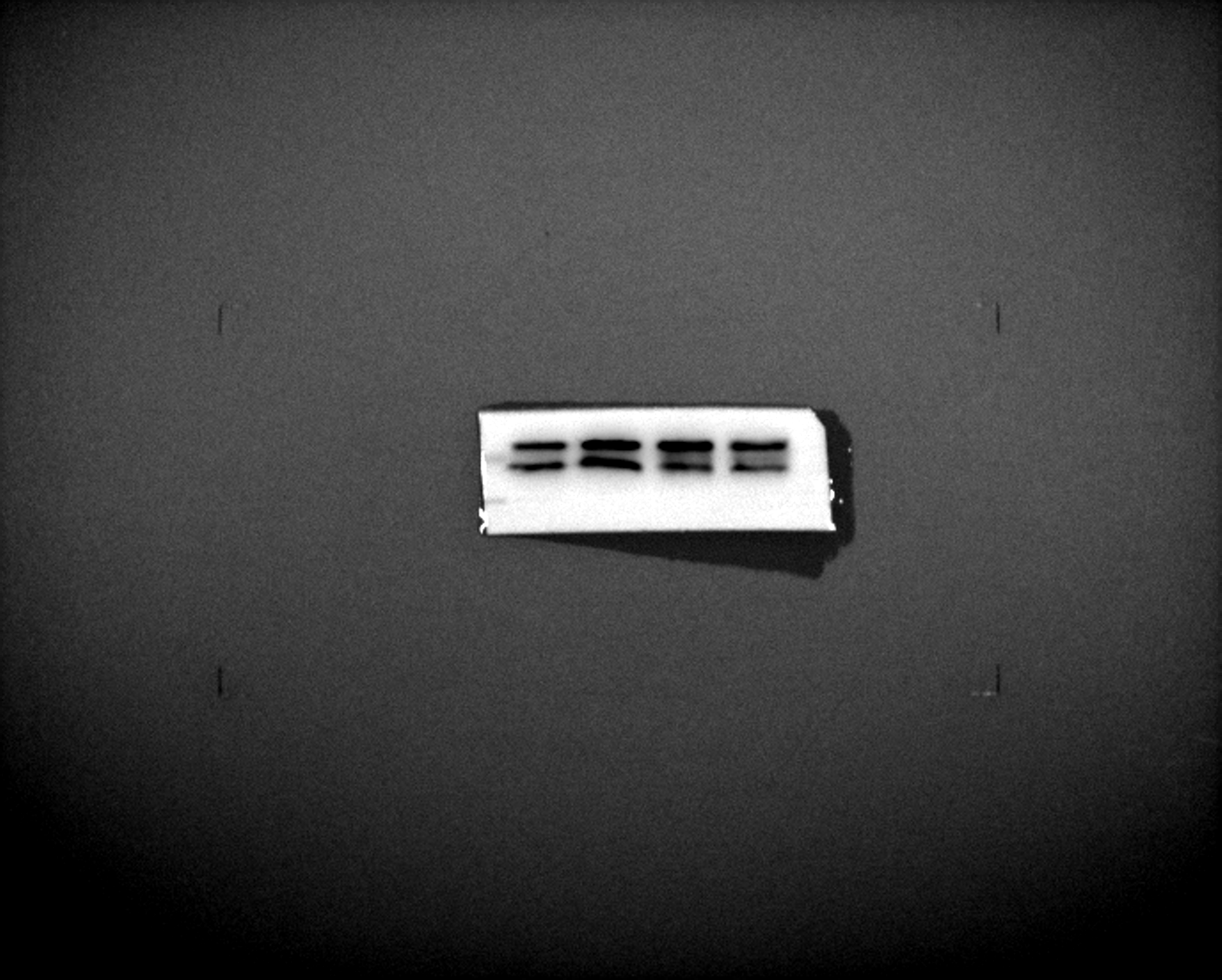

Supplement: Supplementary file 24 [file Image12.TIF]
